# Extracellular Matrix can Recover the Downregulation of Adhesion Molecules after Cell Detachment and Enhance Endothelial Cell Engraftment

**DOI:** 10.1038/srep10902

**Published:** 2015-06-03

**Authors:** Ningning He, Yang Xu, Wei Du, Xin Qi, Lu Liang, Yuebing Wang, Guowei Feng, Yan Fan, Zhongchao Han, Deling Kong, Zhen Cheng, Joseph C. Wu, Zuoxiang He, Zongjin Li

**Affiliations:** 1 Collaborative Innovation Center for Biotherapy, Nankai University School of Medicine, Tianjin, China; 2 Tianjin Key Laboratory of Tumor Microenvironment and Neurovascular Regulation, Nankai University School of Medicine, Tianjin, China; 3 The Key Laboratory of Bioactive Materials, Ministry of Education, College of Life Sciences, Nankai University, Tianjin, China; 4 Department of Cardiology, Tianjin Union Medical Center, Nankai University Affiliated Hospital, Tianjin, China; 5 State Key Lab of Experimental Hematology, Chinese Academy of Medical Sciences, Tianjin, China; 6 Department of Radiology and Molecular Imaging Program at Stanford (MIPS); 7 Stanford Cardiovascular Institute, Stanford University School of Medicine, Stanford, CA, USA; 8 Department of Cardiac Nuclear Imaging, Fuwai Hospital, Peking Union Medical College & Chinese Academy of Medical Sciences, Beijing, China

## Abstract

The low cell engraftment after transplantation limits the successful application of stem cell therapy and the exact pathway leading to acute donor cell death following transplantation is still unknown. Here we investigated if processes involved in cell preparation could initiate downregulation of adhesion-related survival signals, and further affect cell engraftment after transplantation. Human embryonic stem cell-derived endothelial cells (hESC-ECs) were suspended in PBS or Matrigel and kept at 4 °C. Quantitative RT-PCR analysis was used to test the adhesion and apoptosis genes’ expression of hESC-ECs. We demonstrated that cell detachment can cause downregulation of cell adhesion and extracellular matrix (ECM) molecules, but no obvious cell anoikis, a form of apoptosis after cell detachment, was observed. The downregulation of adhesion and ECM molecules could be regained in the presence of Matrigel. Finally, we transplanted hESC-ECs into a mouse myocardial ischemia model. When transplanted with Matrigel, the long-term engraftment of hESC-ECs was increased through promoting angiogenesis and inhibiting apoptosis, and this was confirmed by bioluminescence imaging. In conclusion, ECM could rescue the functional genes expression after cell detached from culture dish, and this finding highlights the importance of increasing stem cell engraftment by mimicking stem cell niches through ECM application.

With their capacity for self-renewal and differentiation, stem cells are promising for the treatment of degenerative diseases or injury, including Type I diabetes mellitus, Parkinson’s disease, Huntington’s disease, myocardial infraction, muscle damage and many others. However, stem cell therapy is limited by low cell retention and engraftment after transplantation^1^,^2^,^3^. For instance, bioluminescence imaging (BLI) data on transplantation of endothelial cells for heart ischemia therapy revealed only 1.5-2.0% survival after 4-8 weeks^4^,^5^,^6^. To overcome low engraftment, the development of a strategy to alleviate apoptotic cell death would be important for stem cell based therapy^7^.

The exact pathways leading to acute donor cell death following transplantation are still unknown, but absence of survival factors, disruption of cell-cell interaction coupled with loss of survival signals from matrix attachments, insufficient vascular supply, and elaboration of inflammatory cytokines resulting from ischemia and/or cell death probably all play major roles^3^,^8^. Traditional stem cell preparations for experimental or clinical transplantation involve enzymatically dispersed cells suspended in phosphate-buffered saline (PBS), stored for minutes to hours on ice at 4 °C. During this period, important adhesion-related survival signals could be absent and a pathway of cell death called “anoikis” (Greek: state of homelessness), a form of apoptosis, will be initiated^9^,^10^,^11^. It is believed that stem cells require a very strictly controlled environment in order to remain viable and healthy from the time of cell processing to transplantation^12^.

Since cell adhesion to matrix is an absolute requirement for survival and proliferation of anchorage dependent cells, the failure to adhere to a substratum may represent a signal to activate a suicide process during storage in suspension before transplantation^13^. The strategy to seed stem cells on synthetic structures that are designed to mimic the extracellular matrix (ECM) before transplantation provides not only a scaffold for cell anchorage, but also a supportive niche for engraftment or accelerating stem cell differentiation. Those materials may reduce the number of stem cells needed for effective tissue reconstitution, as well as promote stem cell self-renewal^7^,^14^,^15^. Many studies have used Matrigel, a reconstituted basement membrane derived from the Engelbroth-Holm-Swarm (EHS) mouse sarcoma that mimics mechanical and biochemical properties of ECM, to facilitate cellular self-organization^16^. Engineered microenvironments with ECM have been increasingly successful in controlling stem cell fate by emulating the key regulatory signals such as survival, growth, differentiation, and migration^17^,^18^,^19^.

Here we hypothesized that when suspended in PBS and stored at 4 °C, the anoikis of human embryonic stem cell-derived endothelial cells (hESC-ECs), will be initiated; whereas suspended in Matrigel will block this process and further enhance cell engraftment after transplanted into mouse myocardial infraction model. To test this hypothesis, we investigated the cell adhesion and apoptosis genes’ expression of hESC-ECs suspended in PBS or Matrigel with RT^2^ Profiler^TM^ PCR Array. We also transplanted hESC-ECs into a mouse myocardial ischemia model and further examined the cell survival with BLI and cardiac function by echocardiogram and pressure-volume (PV) acquisition.

## Results

### Adhesion Molecules Expression after Cell Detachment

**Figure 1:**
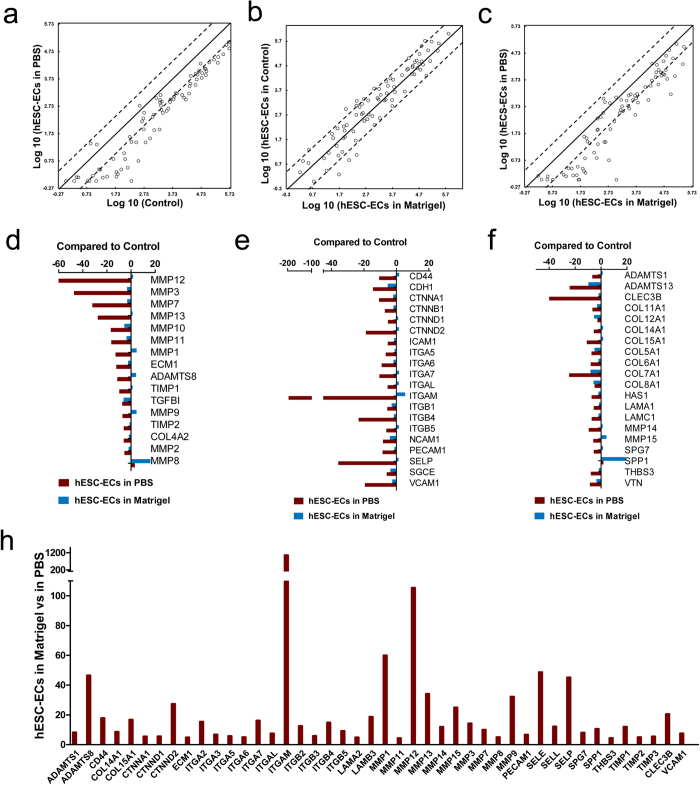
The extracellular matrix (ECM) and cell adhesion related gene-expression patterns of hESC-EC at different conditions. hESC-ECs were released from the substrates and suspended in PBS or Matrigel for 2 h at 4 °C. Total RNA samples were isolated and qRT-PCR assays were performed using PCR Array (Human Extracellular Matrix and Adhesion Molecules RT2 Profiler^TM^ PCR Array). The lines indicate the diagonal and 4-fold changes between the two samples. (**a**) Comparisons between hESC-ECs in PBS and control (hESC-ECs in culture dish), **(b)** between control and hESC-ECs in Matrigel, and (**c**) between hESCs in PBS and hESC-ECs in Matrigel. Expression fold-changes of the genes that are differentially expressed in hESC-ECs in PBS and hESC-ECs in Matrigel compared with hESC-ECs in culture dish (control) focus on (**d**) ECM genes, (**e**) cell adhesion molecules genes, and (**f**) ECM and cell adhesion molecules genes. (**g**) Expression of more than 4-fold changes genes of hESC-ECs in Matrigel compared with hESC-ECs in PBS. Supplemental Tables 1S-3S showed folds change of over-expressed and under-expressed genes. Each group, n = 4.

Traditional cell preparations for transplantation involve enzymatically dispersed cells, suspended in a protein-free medium, and stored for minutes to hours on ice^9^. During this period, important adhesion-related survival signals could be absent and not re-initiated for many hours until the cells find themselves in the context of a recipient tissue; even then, the proper basal surface for the cells may not be present. In order to define the changes of adhesion molecules expression after cell detachment, we performed RT-PCR assays using PCR Array (Human Extracellular Matrix and Adhesion Molecules RT^2^ Profiler^TM^ PCR Array, 84 genes total) on (i) hESC-ECs suspension in PBS, (ii) hESC-ECs suspension in Matrigel, and (iii) cultured hESC-ECs as positive controls (n = 4/group). We performed a comparative analysis of these gene expression data and carried out a functional annotation of the differentially expressed genes between them (Fig. 1). We found that a set of ECM and adhesion molecules-specific genes was markedly downregulated in hESC-ECs suspended in PBS versus cultured hESC-ECs or hESC-ECs suspended in Matrigel. Interestingly, we found no obvious changes between cultured hESC-ECs and hESC-ECs suspended in Matrigel, which indicates that Matrigel can reverse the downregulation of ECM and adhesion molecules after cell detachment (Fig. 1a–c). Furthermore, comparisons of the gene expression fold-change of ECM pathway (Fig. 1d), cell adhesion molecules pathway (Fig. 1e), ECM and cell adhesion molecule pathways (Fig. 1f) revealed marked genes downregulation when hESC-ECs stored in PBS, but Matrigel can rescue this downregulation. Detailed analyses revealed that several gene families, including integrins (ITGA1, ITGA2, ITGA3, ITGA4, ITGA5, ITGA6, ITGA7, ITGA8, ITGAL, ITGAM, ITGAV, ITGB1, ITGB2, ITGB3, ITGB4, ITGB5), MMPs (MMP12, MMP3, MMP7, MMP13, MMP10, MMP11, MMP1, MMP14, MMP15), collagens (COL4A2, COL11A1, COL12A1, COL14A1, COL15A1, COL5A1, COL6A1, COL7A1, COL8A1), cadherins (CTNNA1, CTNNB1, CTNND1 CTNND2), laminins (LAMA1, LAMC1), and a disintegrin and metalloproteinase with thrombospondin motifs (ADAMTS1, ADAMTS13, ADAMTS8) were downregulated when hESC-ECs stored in PBS. MMPs and Integrins expression is less affected in hESC-ECs suspended in Matrigel group (Fig. 1d). Furthermore, there is an apparent downregulation of the transmembrane molecule genes of cell adhesion, such as SELP (Selectin P) and VACM1 (Vasopressin-activated calcium-mobilizing receptor) in the group of hESC-ECs suspended in PBS (Fig. 1e). Among the highly downregulated genes in hESC-ECs suspended in PBS, MMP8, MMP9, ADAMTS8, MMP1, ITGAM, and SPP1 have a higher upregulation after cell suspension in Matrigel (Fig. 1d–g). MMP8, a molecule involved in stem cell mobilization and trafficking^20^, and SPP1, involved in binding to integrins involved in tissue remodeling *in vivo*^21^, have as high as 20-fold upregulation in the presence of Matrigel (Fig. 1d,f). Moreover, a set of ECM and adhesion molecule-specific genes was markedly upregulated in hESC-ECs suspended in Matrigel group compared with hESC-ECs suspended in PBS group (Fig. 1g). These results indicate that Matrigel can provide an optimal microenvironment for cell residing. Functional gene grouping and detailed gene analysis data can be found in Supplemental Method and Result. Functional annotations of the upregulated or downregulated genes can be found in Table 1S-3S.

### Apoptotic Molecules Expression after Cell Detachment

**Figure 2:**
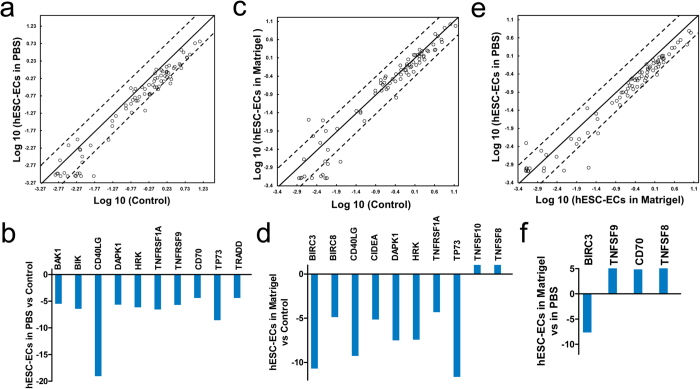
Apoptosis related gene-expression patterns of hESC-ECs at different condition. The lines indicate the diagonal and 4-fold changes between the two groups. (**a**) Comparison between hESC-ECs in PBS and control (hESC-ECs in culture dish), and **(b)** more than 4-fold change genes. **(c)** Comparison between control and hESC-ECs in Matrigel, and **(d)** more than 4 fold change genes. (**e**) Comparison between hESCs in PBS and hESC-ECs in Matrigel, and **(f)** more than 4-fold change genes. Each group (n = 4).

A growing body of evidence is revealing that the binding of a cell to matrix is a key factor for cellular homeostasis, and disruption of such interaction has adverse effects on cell survival^22^. To investigate changes of apoptosis related genes during cell detachment and 4 °C storage, we performed quantitative real-time PCR analysis using the Human Apoptosis PCR array (84 genes total) on (i) hESC-ECs suspension in PBS, (ii) hESC-ECs suspension in Matrigel, and (iii) cultured hESC-ECs as positive control (n = 4/group). Scatter plot analysis demonstrated that no obvious gene expression changes were detected in the group of hESC-ECs in PBS compared to groups of hESC-ECs in Matrigel and control (Fig. 2, Table S4 & S5). Interestingly, apoptosis genes revealed the same expression pattern for cultured hESC-ECs and hESC-ECs suspended in Matrigel (Fig. 2e). More than 4 fold-change genes were listed in Fig. 2b–f. Compared to the marked ECM and cell adhesion genes alteration, fewer gene changes were detected and most apoptosis gene changes were limited to the threshold of 4 folds. Only 10 genes out of 84 were changed in the group of hESC-EC in PBS (Fig. 2b). Apoptosis inducing genes, such as BAK1, BIK, TNFRSF1A, TNFRSF9, HRK, CD70, and TRADD, and anti-apoptosis genes, DAPK1, TP73, and CD40LG, both are downregulated (Fig. 2b). However, most of them were slightly changed (about 5 times) in the group of hESC-ECs in Matrigel. Moreover, 10 genes out of 84 apoptosis inducing genes and anti-apoptosis genes showed changes in hESC-ECs suspended in Matrigel group compared with control (Fig. 2d). Apoptosis inducing genes, TNFSF9, CD70, and TNFSF8, are upregulated, and anti-apoptosis gene BIRC3 is downregulated in hESC-ECs suspended in Matrigel group compared with hESC-ECs suspended in PBS (Fig. 2f). Functional gene grouping and detailed gene analysis data can be found in Supplemental Method and Result. Functional annotations of the upregulated or downregulated genes can be found in Table 4S-6S.

We were intrigued about the relative extent of apoptosis after cell detachment up to 24 hours. We further double stained hESC-ECs with Annexin V (early apoptosis) and propidium iodide (PI) (cell death) 2 and 24 hours after cells suspended in PBS or Matrigel, which were then analyzed with flow cytometry. These results indicate that the storage of hESC-ECs in PBS or Matrigel could induce cell apoptosis after cell detachment, but no significant difference between two groups (Fig. S2). Further trypan blue exclusion assay was consistent with flow cytometry analysis and results revealed slightly cell viability decreasing, but no significant difference between hESC-ECs suspended in PBS and Matrigel (Fig. S3).

### Apoptosis and Angiogenesis Analysis of Early Stage

**Figure 3:**
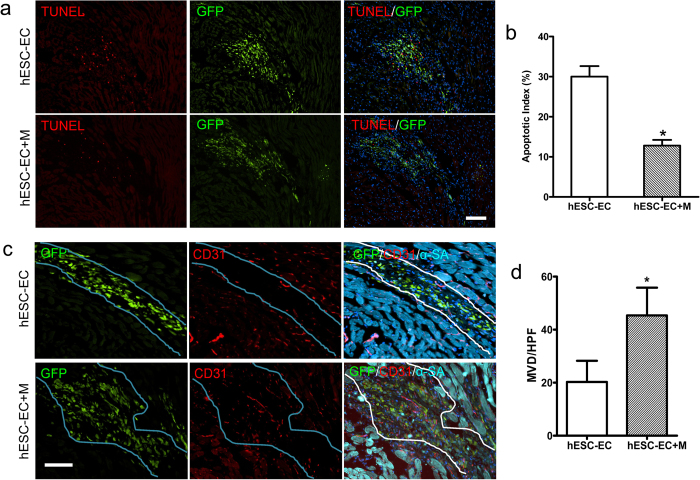
Apoptosis and angiogenesis analysis of early stage after hESC-ECs transplantation. (**a**) Representative photomicrographs showing TUNEL staining in the injected cell clumps at day 3. Cell apoptosis of hESC-EC transplantation (up panel) and hESC-ECs transplanted with Matrigel (hESC-EC + M; lower panel). Green channel shows GFP-labeled graft cells. Red channel shows TUNEL fluorescent staining. Nuclear staining is identified by DAPI (blue). hESC-EC (n = 5), hESC-EC + M (n = 5). (**b**) Quantitative analysis of TUNEL assay. hESC-EC apoptosis were inhibited by Matrigel application. (**c**) Confirmation of hESC-EC engraftment by immunostaining. Clump formation of injected hESC-ECs was observed and immunostaining with GFP (green) and α-sarcomeric actin (α-SA) (Cyan) at day 3. Immunostaining with mouse CD31 (red) revealed more host vasculatures extended into injected cells clumps (Note: mouse CD31 antibody not react with hESC-EC). (**d**) Quantitative analysis revealed increased microvascular density (MVD) per high-power field (HPF) by Matrigel application. The lines indicate the graft region defined by the GFP-labeled graft cells. Nuclear staining is identified by DAPI (blue). ^*^P < 0.05 compared to control. Scale bar = 50 μm. hESC-EC (n = 5), hESC-EC-M (n = 5). hESC-EC + M, hESC-ECs transplanted with Matrigel.

**Figure 4:**
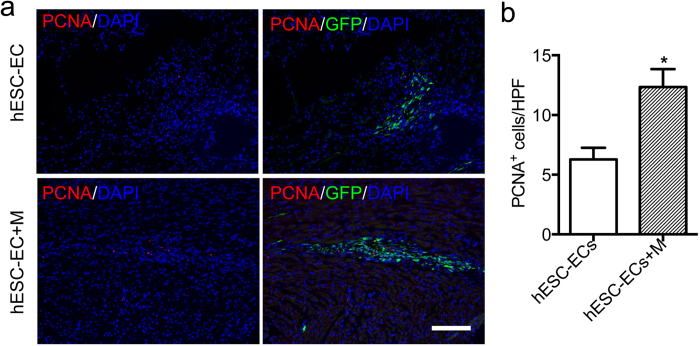
Proliferation analysis after cell transplantation. Monoclonal rat anti-PCNA (red) was used to assess cell proliferation at day 3 after cell transplantation. Quantitative analysis revealed that the number of PCNA^ + ^ cells was markedly higher in heart injected hESC-ECs with Matrigel than those injected hESC-ECs only. *P < 0.05 compared to hESC-ECs group. Scale bar = 50 μm. hESC-EC (n = 5), hESC-EC-M (n = 5). hESC-EC + M, hESC-ECs transplanted with Matrigel.

To understand the therapeutic potential of hESC-ECs for treatment of ischemia heart disease, we subjected adult SCID mice to LAD ischemia-reperfusion followed by injection with 1 × 10^6^ hESC-ECs, or 1 × 10^6^ hESC-ECs in Matrigel, or PBS only as control. Previous studies have revealed that most cell death occurs in the first few days post-transplantation. It is therefore reasonable to assume that cell death mostly occurrs in the first few days after transplantation. To evaluate the cell viability after hESC-EC transplantation, we performed TUNEL staining in the ischemic tissues. Representative photographs of TUNEL-positive nuclei in the transplanted GFP^ + ^hESC-ECs at day 3 are shown in Fig. 3a. The result demonstrated a significant difference in the percentage of apoptotic cells between hESC-ECs group (~30%) and hESC-ECs in Matrigel group (~15%) (Fig. 3b). This suggests that ECM could provide optimal microenvironments in the lesions and further lead to less apoptosis of the engrafted cells. Without adequate host blood supply, the nutrient transportation to the transplanted hESC-ECs clumps is likely inadequate, which could induce cell apoptosis. We thus examined whether Matrigel could increase angiogenesis of transplanted cell clumps in the context of myocardial infarction. Our results revealed that the capillary density at day 3 was significantly greater in hESC-ECs transplanted with Matrigel group compared to hESC-ECs group (Fig. 3c). The increasing angiogenesis will benefit the survival of transplanted cells and may further contribute to functional recovery of heart mediated by local effects. To investigate whether Matrigel promotes cell proliferation *in vivo*, PCNA (proliferating cell nuclear antigen) staining was performed at day 3 after cell transplantation. The number of PCNA^ + ^ cells was markedly higher in hearts injected hESC-ECs with Matrigel than those injected hESC-ECs only. However, PCNA and GFP double positive cells were seldom (Fig. 4), which indicated activation of host cell proliferation, but the proliferation of transplanted cells was limited. To assess if Matrigel could induce angiogenesis *in vivo*, CD31 staining was done at day 56 after Matrigel only injection. Increased angiogenesis was observed (Fig. S4), which is consistent with previous study that local Matrigel injection could promote neoangiogenesis^23^.

### Matrigel Increases Cell Engraftment

**Figure 5:**
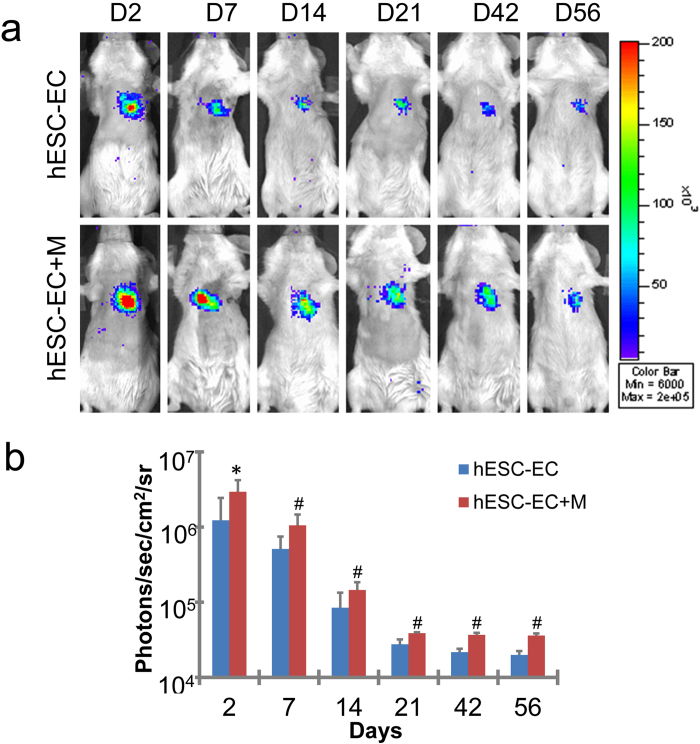
Molecular imaging of hESC-ECs fate after transplantation. (**a**) A representative animal injected with 1 × 10^6^ hESC-ECs alone versus hESC-ECs in Matrigel (hESC-EC + M). (**b**) Quantitative analysis of BLI signals demonstrated that hESC-ECs in Matrigel showed significantly better survival at all time points. *P < 0.05; ^#^*P* < 0.01 compared with hESC-ECs. hESC-EC (n = 10), hESC-EC + M (n = 10).

In order to better understand the long-term cell survival efficacy *in vivo*, we next evaluated the survival of hESC-ECs using non-invasive bioluminescence imaging (BLI). Our BLI data showed that 2 days after intramyocardial transplantation, both hESC-ECs and hESC-ECs in Matrigel groups exhibited robust signals from the cardiac region, thereby confirming successful transplantation. However, in the following days, both groups experienced donor cell death, more significantly in hESC-ECs group (Fig. 5). Serial BLI of the same animals up to 8 weeks demonstrated a significant increasing of cell engraftment with Matrigel application, which suggests that ECM could augment cell survival and might provide a way to increase the number of active cells in ischemic tissues, thereby increasing their therapeutic potential.

### Functional Evaluation of Transplanted hESC-EC

**Figure 6:**
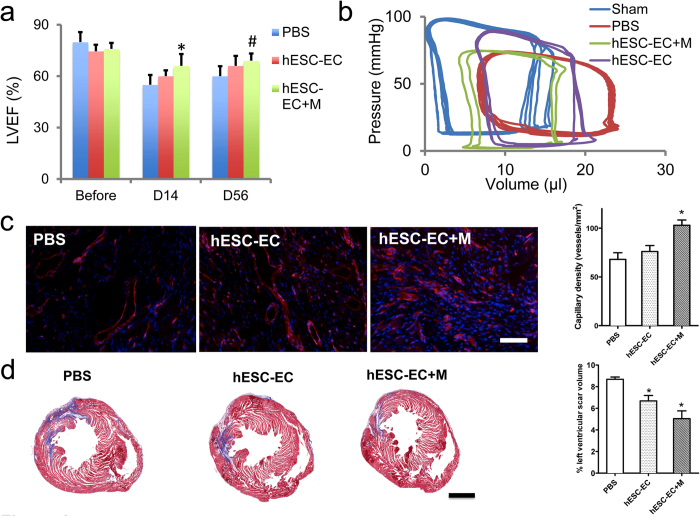
Functional evaluation of transplanted hESC-ECs. (**a**) Comparison of left ventricular ejection fr**a**ction (LVEF) among the 3 groups. Compared with PBS control, hESC-ECs in Matrigel group had significantly higher LVEF at day 14 and 56 (*P < 0.05, ^#^*P* < 0.01 vs PBS group). The hESC-ECs alone group only showed a positive trend toward improvement. (**b**) Representative pressure volume (P-V) loops measured from sham (n = 5), hESC-EC (n = 7), hESC-EC + M (n = 7), or PBS (n = 6) treated at day 56. Curvilinear end-systolic P-V relations in hESC-EC + M treated mice were shifted to the left, indicating enhanced contractility. **(c)** Immunofluorescence staining of CD31 endothelial marker (red) indi**c**ates increased capillary density in the myocardium. Quantitative analysis demonstrated increased capillary density in hESC-EC-M group. Nuclear staining is identified by DAPI (blue). Data are expressed as mean ± SEM. Scale bar = 10 μm. *P < 0.05 vs. PBS group. hESC-EC (n = 9), hESC-EC + M (n = 9), or PBS (n = 7). **(d)** Representative Masson’s trichrome stain of hearts at week 8 showed decrease**d** fibrosis area for hESC-EC and hESC-EC + M groups. Scale bar = 1500μm. *P < 0.05 vs. PBS group. hESC-EC (n = 10), hESC-EC + M (n = 10), or PBS (n = 8). hESC-EC + M, hESC-ECs transplanted with Matrigel.

Echocardiograms were performed at baseline, day 14, and day 56 post transplantation (Fig. 6a). At days 14 and 56, we observed a significant improvement in the left ventricular ejection fraction (LVEF) for the hESC-ECs with Matrigel treated group compared to control group (*P* < 0.01, *P* < 0.05 respectively), but no significant increase was detected for the hESC-EC-treated group compared to the control group. These findings were further corroborated using pressure volume (PV) loops at week 8, which showed a left shifted curvilinear end-systolic P-V loop in hESC-ECs with Matrigel-treated mice (Fig. 6b), indicating enhanced contractility.

### Histological Assessment of Infarction

Histological analysis of the myocardium was performed by examining thin sections of the gross specimen and via immunofluorescent microscopic examination. Examination of the explanted hearts showed an upward trend in microvascular density (MVD) in the hESC-ECs with Matrigel treated group, and these differences were significant compared to hESC-ECs alone and control, (Fig. 6c). Masson trichrome staining indicated a reduced infarction size for the hESC-ECs alone and hESC-ECs with Matrigel compared with PBS control at Day 56 (Fig. 6d).

## Discussion

The salient findings of this work are that: 1) cell detachment can cause downregulation of cell adhesion and ECM molecules, but this will not induce obvious cell anoikis; 2) Matrigel can reverse the downregulation of adhesion and ECM molecules when cells stored in PBS at 4 °C; and 3) Matrigel can further increase heart function by mimicking stem cell niches through promoting angiogenesis and inhibiting apoptosis pathway. In contrast to the previous understanding, our results revealed that hESC-ECs detached from culture flask and kept on ice for several hours will not initiate apparent anoikis (i.e., apoptosis due to the loss of cell anchorage). And no apparent anti-anoikis effect of Matrigel was observed during cell preparation stage *in vitro*, but the disruption of cell anchorage will affect hESC-EC engraftment after transplantation and Matrigel application could rescue this processes.

Ischemic heart disease is a leading cause of mortality and morbidity worldwide. Stem or progenitor cells, including skeletal muscle derived stem cells, cardiomyocytes, mesenchymal stem cells (MSCs) and endothelial cells have shown their beneficial effects after myocardial infarction^8^,^24^. Therapeutic angiogenesis, with endothelial/progenitor cells transplantation is a promising and novel strategy to rescue ischemic myocyte damage, enhance vascular density and promote the regeneration of ischemic tissues^25^. When endothelial cells are prepared for transplantation as single cells, several procedures will be involved. For experimental studies, stem cells will be trypsinized and held on ice for a while. For clinical application, stem cells need to be transported and made available as an off-the-shelf product from companies to market^26^. Freshly harvested or cultured stem cells will be in solutions for hours. Previous study has revealed that when cells being prepared for transplantation as single cells, integrin-ECM interactions were lost and apoptosis was initiated^7^. Furthermore, the recipient tissue *in vivo* is in stark contrast to *in vitro* culture systems, which also could trigger cell apoptosis^6^,^27^,^28^.

Previous research has demonstrated that when adhesion is prohibited, endothelial cells rapidly undergo cell death with the morphological and biochemical characteristics of apoptosis^29^. Our results revealed that no apparent anoikis was initiated when the cell suspension was kept in PBS on ice for 2 to 24 hours, corroborating with previous reports on human mesenchymal stem cells (MSCs)^30^. At present, we can only speculate that the discrepancies in findings may be attributed to differences in cell type or isolation methods^26^,^28^. However, the marked downregulation of adhesion molecules may initiate the further anoikis *in vivo* after cell transplantation. Moreover, others have shown that cold storage alone could cause a decrease in cell viability, especially after 48 hours^10^. BLI data from our study suggest that by day 56, less 1% of the transplanted hESC-ECs are still alive even in co-transplanted with Matrigel. The pattern of donor cell death in this study is consistent with previous reports^4^,^5^,^27^. A number of culprits may be involved, including the loss of cell anchorage occurring during cell preparation prior to transplantation and the stress imposed upon hESC-ECs within a fast-beating ischemic heart. Furthermore, the clumps formation of transplanted cells after intramyocardial injection, as confirmed by histology, is in stark contrast to the rich nutrients and oxygen concentration that have been optimized for their cell culturing, growth, and expansions *in vitro*. To counteract the major pathways of cell death after cell preparation *in vitro* and after transplantation *in vivo*, two general strategies can be employed. The first is by targeting a specific molecular pathway *in vitro*, including the activation of an anti-apoptotic pathway. The second is by inducing a broader spectrum of cytoprotective state *in vivo*, such as the enhancement of nutrient supply for transplanted cell by promoting angiogenesis.

Low cell retention and engraftment after transplantation is a key factor limiting the successful application of stem cell therapy^1^. The special stem cell microenvironments are also known as “niches”, where stem cells integrate a complex array of molecular signals controlling their function and hemostasis^31^. The enhancement of supportive niche function with ECM during transplantation could provide a novel and effective strategy^2^. In this study, our results revealed that gene expressions related to intergrin, MMP, collagen, cadherin, laminin, a disintegrin and metalloproteinase with thrombospondin motifs (ADAMTS) were downregulated after cell detachment from culture plate. Our results are consistent with previous reports that integrin and MMP have profound effects on cell survival^22^,^32^,^33^. However, Matrigel is a solubilized basement membrane preparation extracted from EHS mouse sarcoma and not compatible with clinical applications. The development of effective techniques to mimic the *in vivo* environment and to engineer matrices for activating the pathways, such as integrin, MMP, collagen, cadherin and laminin, is promising for translational application of stem cells. This has been confirmed that the addition of short peptides from the structure of laminin (YIGSR or IKVAV) could promote cell surviving^7^. Furthermore, appropriate modifications of signals from the niche could promote stem cell engraftment or accelerate stem cell differentiation, and perhaps reduce the number of stem cells needed for effective tissue reconstitution and promote stem cell self-renewal^14^. As therapies advance from research to clinical applications, stem cell therapy could be significantly and economically improved by optimizing storage conditions and creating an ideal scaffold for cell anchorage^13^,^20^.

In conclusion, our results here show that hESC-ECs will lose cell adhesion and ECM molecules after enzymatic digestion and keeping in PBS, but are also able to regain or recover those molecules expression in the presence of Matrigel, a kind of ECM. ECM therefore has been shown to rescue the functional genes expression after cell detached from culture dish, providing a new method to increase efficiency of stem cell therapy, which if proven could lead to substantial changes in stem cell administration. Importantly, our study on hESC-based therapy showed increasing long-term engraftment of hESC-ECs in Matrigel by serial BLI. Thus, to sustain long-term engraftment of stem cells, alternative transplantation protocols, including the addition of matrix to set up artificial stem cell niches to prevent cells death after transplantation demonstrated here, may prove to be more viable approaches.

## Materials and Methods

### Cell Culture

Undifferentiated human embryonic stem cells (hESCs) (H9 line) were purchased from Wicell Research Institute (Madison, WI, USA). Differentiation of hESCs to endothelial cells was performed using the two-step protocol we described previously^5^. Briefly, we cultured hESCs in ultra-low attachment plates (Corning, Lowell, MA) to form embryoid bodies (EBs), the three-dimensional aggregates of hESCs. Next, we harvested 12-day-old EBs, seeded them in rat-tail collagen type I (BD Biosciences, Bedford, MA), and obtained EB sproutings 3 days later. CD31^+^/CD144^+^ cells of EB-sprouting were isolated as hESC derived endothelial cells (hESC-ECs) via flow cytometry as described^5^, and cultured in EGM-2 medium (Lonza, Walkersville, MD). To track transplanted cells *in vivo*, hESC-ECs were transduced with a self-inactivating lentiviral vector carrying an ubiquitin promoter driving firefly luciferase and green fluorescence protein (Fluc-GFP) double-fusion (DF) reporter gene^4^,^34^.

### Cell Suspension Preparation

hESC-ECs were released from the substrates using a solution of 0.05 (w/v)% trypsin/0.53 mM EDTA (Invitrogen, Carlsbad, CA). The harvested cells were centrifuged, and 1.0 × 10^6^ cells were suspended in 20 μl of phosphate-buffered saline (PBS; Invitrogen) or Matrigel (BD Pharmingen, San Jose, CA) and stored for 2 h at 4 °C. Total RNA samples were isolated with Trizol (Invitrogen), followed by purification over a Qiagen RNeasy column (Qiagen, Waltham, MA) from hESC-ECs in culture dish, hESC-ECs in PBS, and hESC-ECs in Matrigel (hESC-EC + M). Four samples from each group (for a total of 12 unique samples) were harvested for RNA isolation. An outline of the experiments is depicted in Fig. S1.

### Quantitative RT-PCR (qRT-PCR)

qRT-PCR assays were performed using human ECM and Adhesion Molecules PCR Array (PAHS-013; SuperArray Bioscience, Frederick, MD) and human apoptosis PCR Array (PAHS-012; SuperArray Bioscience) on an ABI PRISM 7900 HT (Applied Biosystems, Foster City, CA). Data analysis is available at the company website (http://www.superarray.com/pcr/arrayanalysis.php). First-strand cDNAs were generated using iScript Select cDNA Synthesis Kit (BioRad, Hercules, CA). For real-time PCR reaction, first-strand cDNAs were added to RT qPCR Master Mix (SuperArray Bioscience). Samples were heated for 10 min at 95 °C and then subjected to 40 cycles of denaturation at 95 °C for 15 sec and annealing and elongation at 60 °C for 1 min.

### Cell Apoptosis Analysis

For apoptosis analysis, cells were prepared using Annexin V-fluorescein isothiocyanate (FITC) apoptosis detection kit (BD Pharmingen) according to the manufacturer’s directions, and stained cells were analyzed by FACS. Trypan blue exclusion assay was used to assess cell viability. Six samples were performed and results were averaged.

### Transplantation of hESC-ECs into Ischemia Myocardium

All surgical procedures were performed on 8–10 week old female SCID Beige mice (Laboratory Animal Center of the Academy of Military Medical Science, Beijing, China) by a single experienced micro-surgeon. Protocols were approved by the Nankai University Animal Care and Use Committee guidelines, which conform to the Guide for the Care and Use of Laboratory Animals published by the US National Institutes of Health (8^th^ edition, 2011). Following induction with inhaled isoflurane (2% to 3%), mice were intubated and ventilated and anesthesia was maintained with inhaled isoflurane (1% to 2.5%). During the operation, the heart rate, respiratory rate, and body temperature of the mice were carefully monitored. A left thoracotomy was performed, followed by ligation of mid-left anterior descending (LAD) artery for 30 minutes, and then followed by reperfusion. Infarction was visually confirmed by blanching of the anterolateral region of the left ventricle along with dyskinesis. After 30 minutes, 1 × 10^6^ hESC-ECs in either PBS or Matrigel were injected intramyocardially into the peri-infarct zone at 20 μl of total volume (n = 15 each). Control animals received PBS (n = 15) or Matrigel (n = 5) injection instead. For sham-operated animals, without ischemia/reperfusion surgery (n = 5), suturing was performed without ischemia-reperfusion (I/R). After surgery, buprenorphine (0.05/mg/kg, s.c.) was applied every 12 hours for 3 days for pain relief. At end of experiment, mice were sacrificed by exposure to 100% CO_2_ followed by cervical dislocation, and heart samples were harvested for appropriate analysis.

### Bioluminescence Imaging of Transplanted Cell

Cardiac bioluminescence imaging (BLI) was performed on all animals using the Xenogen IVIS Lumina II system by a blinded investigator (YX). A majority of the animals were scanned on days 2, 7, 14, 21, 42, and 56. After intraperitoneal injection of the reporter probe D-Luciferin (150 mg/kg), animals were imaged for 1 and 2 minutes on day 2 and 7, and 3–5 minutes on day 14 and 21, and 10 minutes on day 42 and 56. The same mice were imaged longitudinally for up to 8 weeks. Bioluminescence signal was quantified in units of maximum photons per second per cm square per steradian (photons/sec/cm^2^/sr) as described^4^.

### Heart Function Analysi**s**

Animals were scanned on baseline (day -7) and days 14 and 56 post-operation using the Siemens-Acuson Sequoia C512 system equipped with a multi-frequency (8-14 MHz) 15L8 transducer. Animals were anesthetized with inhaled 2% isoflurane. Analysis of M-mode images was performed^5^. At the end of the study (day 56), invasive hemodynamic measurements were performed as described^27^. Briefly, after midline neck incision, a conductance 1.4 conductance catheter (Millar Instruments, Houston, TX, USA) was introduced into the left ventricle through the right carotid artery. After stabilization, the signals were continuously recorded at a sampling rate of 1000/s using pressure-volume (PV) conductance system coupled to a PowerLab/4SP analog to digital converter (ADInstruments). Data were analyzed by using a cardiac pressure-volume analysis program (PVAN 3.4; Millar Instruments, Houston, TX, USA) and Chart/Scope Software (AD Instruments, Colorado Springs, CO, USA).

### Histological Analysis

Explanted hearts from study and control groups were embedded into OCT compound (Miles Scientific). Frozen sections (5 μm thick) were processed for immunostaining. To track GFP^+^ hESC-EC in hearts, rabbit anti-GFP antibody (Invitrogen) was used. To investigate angiogenesis after hESC-ECs transplantation, rat anti-mouse CD31 antibody (BD Pharmingen) were used. Anti-α-sarcomeric actin (α-SA) antibody (Sigma) was used for cardiomyocytes staining. Alexa Fluor 488, Alexa Fluor 594, and Alexa Fluor 647-conjugated secondary antibodies were applied. DAPI was used for nuclear counterstaining. To detect microvascular density (MVD) in the peri-infarct area, a rat anti-mouse CD31 (BD Pharmingen) was used. The number of capillary vessels was counted by a blinded investigator (YX) in ten randomly selected areas using a fluorescence microscope (200x magnification). To measure circumferential fibrosis area, Masson’s trichrome staining was performed^35^. Explanted hearts from study and control groups were harvested after euthanasia at 8 weeks after ischemia reperfusion for assessment. Images were taken for each section to calculate the fibrotic and nonfibrotic areas. Scarring was determined by fibrotic area /(fibrotic + nonfibrotic area). The measurement of area of fibrosis was determined by Image J software (NIH) performed on 3 separate sections, and the averages were used for statistical analysis. To investigate cell apoptosis after hESC-ECs transplantation, *in situ* fluorescent TUNEL staining was done according to the manufacturer’s protocol (Chemicon International Inc). Results were analyzed by fluorescent microscopy (Olympus) and quantified with Image J (NIH).

### Statistical Analysis

Statistics were calculated using SPSS 16.0 (SPSS Inc., Chicago, IL, USA). Descriptive statistics included mean and standard error. Two-way repeated measures ANOVA and two-tailed Student’s *t*-test were used. Differences were considered significant at *P* values of < 0.05.

## Additional Information

**How to cite this article**: He, N. *et al*. Extracellular Matrix can Recover the Downregulation of Adhesion Molecules after Cell Detachment and Enhance Endothelial Cell Engraftment. *Sci. Rep*. **5**, 10902; doi: 10.1038/srep10902 (2015).

## Supplementary Material

Supplementary Information
