# Supplementary material for: Extracellular Matrix can Recover the Downregulation of Adhesion Molecules after Cell Detachment and Enhance Endothelial Cell Engraftment
Source: Sci Rep. 2015 Jun 3;5:10902. doi: 10.1038/srep10902 (PMC4454140; doi:10.1038/srep10902)
Supplement: Supplementary Information [file srep10902-s1.pdf]

Extracellular Matrix can Recover the Downregulation of Adhesion Molecules after Cell Detachment and Enhance Endothelial Cell Engraftment

Ningning He, Yang Xu, Wei Du, Xin Qi, Lu Liang, Yuebing Wang, Guowei Feng, Yan Fan, Zhongchao Han, Deling Kong, Zhen Cheng, Joseph C. Wu, Zuoxiang He, Zongjin Li

Supplemental Methods and Results

Supplemental Methods

Matrigel

The concentrations of Matrigel used in this study are between 9-12mg/ml upon specific lot (http://bd.com/resource.aspx?IDX=17841). The concentrations we used in this study are around 10mg/ml and the finial concentrations for injection and in vitro analysis are around 5mg/ml. Moreover, Matrigel was kept on ice all time w/o loaded cells.

Functional Gene Grouping

The functional gene grouping of PCR array, human Extracellular Matrix and Adhesion Molecules RT^2 Profiler^{TM} PCR Array (PAHS-013; SuperArray Bioscience, Frederick, MD), can be found at website: http://www.sabiosciences.com/rt_pcr_product/HTML/PAHS-013Z.html#function, and was listed below:

Cell Adhesion Molecules:

Transmembrane Molecules: CD44, CDH1, HAS1, ICAM1, ITGA1, ITGA2, ITGA3, ITGA4, ITGA5, ITGA6, ITGA7, ITGA8, ITGAL, ITGAM, ITGAV, ITGB1, ITGB2,

ITGB3, ITGB4, ITGB5, MMP14, MMP15, MMP16, NCAM1, PECAM1, SELE, SELL, SELP, SGCE, SPG7, VCAM1.

Cell-Cell Adhesion: CD44, CDH1, COL11A1, COL14A1, COL6A2, CTNND1, ICAM1, ITGA8, VCAM1.

Cell-Matrix Adhesion: ADAMTS13, CD44, ITGA1, ITGA2, ITGA3, ITGA4, ITGA5, ITGA6, ITGA7, ITGA8, ITGAL, ITGAM, ITGAV, ITGB1, ITGB2, ITGB3, ITGB4, ITGB5, SGCE, SPP1, THBS3.

Other Adhesion Molecules: CNTN1, COL12A1, COL15A1, COL16A1, COL5A1, COL6A1, COL7A1, COL8A1, VCAN, CTGF, CTNNA1, CTNNB1, CTNND2, FN1, KAL1, LAMA1, LAMA2, LAMA3, LAMB1, LAMB3, LAMC1, THBS1, THBS2, CLEC3B, TNC, VTN.

Extracellular Matrix Proteins:

Basement Membrane Constituents: COL4A2, COL7A1, LAMA1, LAMA2, LAMA3, LAMB1, LAMB3, LAMC1, SPARC.

Collagens & ECM Structural Constituents: COL11A1, COL12A1, COL14A1, COL15A1, COL16A1, COL1A1, COL4A2, COL5A1, COL6A1, COL6A2, COL7A1, COL8A1, FN1, KAL1.

ECM Proteases: ADAMTS1, ADAMTS13, ADAMTS8, MMP1, MMP10, MMP11, MMP12, MMP13, MMP14, MMP15, MMP16, MMP2, MMP3, MMP7, MMP8, MMP9, SPG7, TIMP1.

ECM Protease Inhibitors: COL7A1, KAL1, THBS1, TIMP1, TIMP2, TIMP3.

Other ECM Molecules: VCAN, CTGF, ECM1, HAS1, SPP1, TGFBI, THBS2, THBS3, CLEC3B, TNC, VTN.

The functional gene grouping of PCR array human apoptosis RT^2 Profiler^{TM} PCR Array (PAHS-012; SuperArray Bioscience), can be found at website: http://www.sabiosciences.com/rt_pcr_product/HTML/PAHS-012A.html#function, and was listed below:

Induction of Apoptosis:

Death Domain Receptors: CRADD, FADD, TNF, TNFRSF10B (DR5).

DNA Damage: ABL1, CIDEA, CIDEB, TP53, TP73.

Extracellular Signals: CFLAR (CASPER), DAPK1, TNFRSF25 (DR3).

Other: BAD, BAK1, BAX, BCL10, BCL2L11, BCLAF1, BID, BIK, BNIP1, BNIP3, BNIP3L, CASP1 (ICE), CASP10 (MCH4), CASP14, CASP2, CASP3, CASP4, CASP6, CASP8, CD27 (TNFRSF7), CD70 (TNFSF7), DFFA, FAS (TNFRSF6), FASLG (TNFSF6), GADD45A, HRK, LTA (TNFB), NOD1 (CARD4), PYCARD (TMS1/ASC), TNFRSF10A, TNFRSF9, TNFSF10 (TRAIL), TNFSF8, TP53BP2, TRADD, TRAF3.

Anti-Apoptosis:

AKT1, BAG1, BAG3, BAG4, BAX, BCL2, BCL2A1 (Bfl-1/A1), BCL2L1 (BCL-X), BCL2L10, BCL2L2, BFAR, BIRC3 (c-IAP1), BIRC6, BIRC8, BNIP1, BNIP2, BNIP3, BNIP3L, BRAF, CD27 (TNFRSF7), CD40LG (TNFSF5), CFLAR (CASPER), DAPK1,

FAS (TNFRSF6), HRK, IGF1R, MCL1, NAIP (BIRC1), NOL3, RIPK2, TNF, XIAP (BIRC4).

Regulation of Apoptosis:

Negative Regulation: BAG1, BAG3, BAG4, BCL10, BCL2, BCL2A1 (Bfl-1/A1), BCL2L1 (BCL-X), BCL2L10, BCL2L2, BFAR, BIRC3 (c-IAP1), BIRC6, BIRC8, BNIP1, BNIP2, BNIP3, BNIP3L, BRAF, CASP3, CD27 (TNFRSF7), CD40LG (TNFSF5), CFLAR (CASPER), CIDEA, DAPK1, DFFA, FAS (TNFRSF6), IGF1R, MCL1, NAIP (BIRC1), NOL3, TP53, TP73, XIAP (BIRC4).

Positive Regulation: ABL1, AKT1, BAD, BAK1, BAX, BCL2L11, BCLAF1, BID, BIK, BNIP3, BNIP3L, CASP1 (ICE), CASP10 (MCH4), CASP14, CASP2, CASP4, CASP6, CASP8, CD70 (TNFSF7), CIDEB, CRADD, FADD, FASLG (TNFSF6), HRK, LTA (TNFB), NOD1 (CARD4), PYCARD (TMS1/ASC), RIPK2, TNF, TNFRSF10A, TNFRSF10B (DR5), TNFRSF25 (DR3), TNFRSF9, TNFSF10 (TRAIL), TNFSF8, TP53, TP53BP2, TRADD, TRAF2, TRAF3, TRAF4.

TNF/TNFR Domain Proteins:

CD27 (TNFRSF7), CD40 (TNFRSF5), FAS (TNFRSF6), FASLG (TNFSF6), LTA (TNFB), LTBR, TNF, TNFSF10 (TRAIL), TNFSF8, TNFRSF10A, TNFRSF10B (DR5), TNFRSF11B, TNFRSF21, TNFRSF25 (DR3), TNFRSF9.

BCL2 and BAG Domain Proteins:

BAG1, BAG3, BAG4, BAX, BCL2, BCL2A1 (Bfl-1/A1), BCL2L1 (BCL-X), BCL2L10, BCL2L11, BCL2L2, MCL1.

BIR Domain Proteins:

BIRC2 (c-IAP2), BIRC3 (c-IAP1), BIRC6, BIRC8, NAIP (BIRC1), XIAP (BIRC4).

CARD Domain Proteins:

APAF1, BCL10, BIRC2 (c-IAP2), BIRC3 (c-IAP1), CARD6, CARD8, CASP1 (ICE), CASP2, CASP4, CASP5, CASP9, CRADD, NOD1 (CARD4), NOL3, PYCARD (TMS1/ASC), RIPK2.

DEATH Domain Proteins:

CRADD, DAPK1, FADD, TNFRSF10A, TNFRSF10B (DR5), TNFRSF11B, TNFRSF1A, TNFRSF21, TNFRSF25 (DR3), TRADD.

TRAF Domain Proteins:

TRAF2, TRAF3, TRAF4.

Caspases and Regulators:

<u>Caspases:</u> CASP1 (ICE), CASP10 (MCH4), CASP14, CASP2, CASP3, CASP4, CASP5, CASP6, CASP7, CASP8, CASP9, CFLAR (CASPER), CRADD, PYCARD (TMS1/ASC).

<u>Caspase Activators:</u> APAF1, BAX, BCL2L10, CARD8, CASP1 (ICE), CASP9, NOD1 (CARD4), PYCARD (TMS1/ASC), TNFRSF10A, TNFRSF10B (DR5), TP53.

<u>Caspase inhibitors:</u> CD27 (TNFRSF7), XIAP (BIRC4).

Supplemental Results

Extracellular Matrix and Adhesion Molecules PCR Array (PAHS-013) full gene list and data analysis results can be found in below table:

		AVG Delta(Ct) (Ct(GOI) - Ave Ct(HKG))			Standard Deviation			2^(-Avg.(Delta(Ct))			p-value (comparing to control group)	
**Position**	**Symbol**	**Control Group**	**hESC-EC in Matrigel**	**hESC-EC in PBS**	**Control Group**	**hESC-EC in Matrigel**	**hESC-EC in PBS**	**Control Group**	**hESC-EC in Matrigel**	**hESC-EC in PBS**	**hESC-EC in Matrigel**	**hESC-EC in PBS**
A01	ADAMTS1	-9.047726	-9.318289	-6.278308	2.275656	0.875084	1.668681	529.220939	638.38768	77.617389	0.483159	0.255788
A02	ADAMTS13	-9.766062	-6.479078	-5.166239	4.808787	2.042859	0.980831	870.717957	89.20654	35.908152	0.134049	0.282573
A03	ADAMTS8	-3.511691	-5.53973	0	3.430631	3.344695	0	11.40576	46.518418	1	0.54119	0.225022
A04	CD44	-16.255284	-17.005006	-12.847374	1.248493	1.276526	0.68395	78221.84801	131527.596	7369.614809	0.417314	0.09506
A05	CDH1	-4.276756	-1.858662	-0.426453	0.928722	1.104767	0.852907	19.383484	3.626712	1.343926	0.010605	0.032097
A06	CNTN1	-1.235643	-0.742943	-0.475077	2.140196	1.716833	0.950155	2.354862	1.673586	1.389993	0.917594	0.379053
A07	COL11A1	-2.737387	-1.128124	0	2.741302	1.930827	0	6.668613	2.185743	1	0.23923	0.211567
A08	COL12A1	-11.135951	-8.694903	-9.62844	0.993281	1.274734	0.620614	2250.377524	414.406469	791.497235	0.02582	0.125849
A09	COL14A1	-3.13305	-3.736128	-0.636756	0.934927	2.94536	1.273512	8.772879	13.325595	1.554829	0.309808	0.054906
A10	COL15A1	-8.151636	-8.763398	-4.694741	1.003078	1.688102	1.216319	284.372143	434.55604	25.897501	0.327412	0.056585
A11	COL16A1	-10.048562	-8.170415	-7.755394	0.894627	1.17711	0.507055	1059.054902	288.097833	216.075846	0.031863	0.056096
A12	COL1A1	-16.647252	-14.302011	-14.403371	0.969119	1.276582	0.570393	102641.3363	20199.20051	21669.39118	0.020813	0.072801
B01	COL4A2	-17.685702	-16.762637	-15.074365	0.777946	0.88053	0.529859	210827.2268	111187.626	34501.35733	0.17886	0.031651
B02	COL5A1	-18.279377	-15.937836	-15.380591	1.075238	1.313537	0.807454	318156.4844	62772.12501	42659.84194	0.025975	0.069134
B03	COL6A1	-16.327917	-15.307983	-13.320408	0.804061	0.671779	0.336218	82260.75352	40565.98784	10229.21808	0.081587	0.028654
B04	COL6A2	-12.758803	-12.501234	-10.660085	1.099617	0.54383	0.497109	6930.783159	5797.576125	1618.099658	0.434901	0.088784
B05	COL7A1	-10.869768	-7.834589	-6.24592	4.863259	1.49494	0.657087	1871.225441	228.268639	75.894333	0.133254	0.282478
B06	COL8A1	-12.556764	-10.000817	-10.230534	1.655966	1.032443	0.492707	6025.07576	1024.580057	1201.427811	0.06401	0.184502
B07	VCAN	-10.486028	-8.506576	-10.081934	0.71659	2.623727	0.423243	1434.197176	363.692624	1083.838812	0.504042	0.300645
B08	CTGF	-15.634022	-14.839687	-14.253391	0.769182	1.24948	0.730955	50852.16817	29321.81932	19529.81618	0.371657	0.098709
B09	CTNNA1	-15.072328	-14.113543	-11.652288	0.939012	0.580606	0.641788	34452.67385	17725.54928	3218.757113	0.097878	0.044894
B10	CTNNB1	-14.361935	-13.479954	-11.523096	0.795099	0.700329	0.58535	21055.86708	11425.37242	2943.048802	0.106495	0.022582
B11	CTNND1	-15.293796	-15.389207	-12.911386	1.268776	1.368026	0.602524	40169.03919	42915.38396	7703.96936	0.816378	0.123592
B12	CTNND2	-4.78249	-5.321427	-0.54816	4.474115	3.772509	1.09632	27.521547	39.986101	1.46222	0.709358	0.232081
C01	ECM1	-13.737169	-12.39834	-10.125081	1.685122	1.268608	1.350862	13655.2553	5398.489729	1116.742422	0.166915	0.169198
C02	FN1	-19.963253	-19.30568	-18.905917	0.596452	0.825487	0.538742	1022204.677	648020.4683	491188.2418	0.221868	0.081435
C03	HAS1	-7.618383	-6.566971	-4.746411	3.080316	2.441455	0.880656	196.499705	94.810269	26.841828	0.285514	0.268832
C04	ICAM1	-11.08073	-10.546664	-8.662439	1.356336	0.903804	0.835089	2165.868093	1495.761044	405.185644	0.342794	0.138301
C05	ITGA1	-10.939484	-11.257834	-9.830937	0.930874	0.625999	0.820955	1963.870385	2448.75644	910.766237	0.679968	0.209849
C06	ITGA2	-12.137073	-13.837863	-9.892274	1.010832	1.738289	0.572265	4504.255656	14642.38703	950.323075	0.345644	0.080592
C07	ITGA3	-15.093333	-15.775196	-13.012884	1.111667	1.255807	0.584709	34957.95894	56079.91058	8265.486371	0.417928	0.106592
C08	ITGA4	-12.896661	-11.724229	-10.616169	1.388456	0.932873	0.493215	7625.73492	3383.331242	1569.586965	0.133798	0.146693
C09	ITGA5	-15.824672	-15.634414	-13.103557	1.189293	1.221864	0.946238	58036.4505	50865.99903	8801.643665	0.86521	0.104036
C10	ITGA6	-11.699337	-10.824187	-8.508342	1.331629	0.609336	0.535599	3325.457997	1813.029358	364.138065	0.144076	0.099112
C11	ITGA7	-6.762678	-7.389786	-3.370595	1.605038	3.990319	0.816071	108.584798	167.705461	10.343091	0.525074	0.153679
C12	ITGA8	-7.397565	-6.196065	-5.363593	2.175562	1.070176	1.102998	168.612186	73.316441	41.172032	0.171815	0.23547
D01	ITGAL	-3.859673	-4.328826	-1.415781	3.663894	2.211896	2.831562	14.517012	20.095854	2.668041	0.587037	0.343315
D02	ITGAM	-7.617345	-10.042677	0	4.059876	4.12688	0	196.358286	1054.743879	1	0.367228	0.261823
D03	ITGAV	-14.521131	-14.716202	-12.765929	0.661379	1.991002	0.677087	23512.34262	26916.48551	6965.105548	0.483478	0.024033
D04	ITGB1	-17.211533	-15.648742	-14.720799	1.246568	0.611054	0.418712	151770.5836	51373.69221	27002.37622	0.060802	0.113368
D05	ITGB2	-6.875666	-6.307769	-2.662228	1.515837	1.10304	1.846796	117.430742	79.218693	6.330098	0.403151	0.133586
D06	ITGB3	-8.83048	-9.777377	-7.233705	0.901913	0.889177	1.056844	455.238996	877.574277	150.508875	0.217453	0.115234
D07	ITGB4	-9.238941	-8.587855	-4.697135	2.652003	2.934413	1.149503	604.224414	384.770626	25.940502	0.971056	0.23856
D08	ITGB5	-14.471012	-15.025527	-11.831395	1.453394	1.396201	0.710362	22709.56315	33352.95578	3644.221032	0.585771	0.147426
D09	KAL1	-9.087799	-8.45799	-8.304133	0.700402	1.354658	0.881473	544.126771	351.648369	316.077165	0.893794	0.275009
D10	LAMA1	-2.513116	-1.801211	0	0.893279	1.521005	0	5.708516	3.485127	1	0.754552	0.035191
D11	LAMA2	-11.044786	-11.441909	-9.166487	1.34322	1.81737	0.53777	2112.573763	2782.0036	574.629202	0.540527	0.158811
D12	LAMA3	-8.561314	-10.723992	-9.139457	1.136701	2.221006	1.072124	377.756909	1691.388246	563.963311	0.482399	0.480078
E01	LAMB1	-15.431982	-14.771083	-13.341007	1.211373	0.830366	0.624716	44206.84029	27960.11578	10376.32175	0.244706	0.127531
E02	LAMB3	-10.193035	-12.274583	-8.051936	0.909955	1.440675	1.408822	1170.602054	4954.701991	265.383663	0.290583	0.132958
E03	LAMC1	-15.198429	-14.246765	-12.333741	0.714197	0.59916	0.516724	37599.58193	19440.33266	5162.094188	0.053311	0.016818
E04	MMP1	-14.301904	-16.517736	-10.611897	1.889094	1.939386	0.371484	20197.71312	93828.33213	1564.945283	0.309247	0.178182
E05	MMP10	-10.246519	-7.763451	-6.175568	2.680561	0.936263	0.740761	1214.813671	217.286002	72.282199	0.113866	0.236504
E06	MMP11	-13.927889	-12.010591	-9.887809	1.968764	1.284712	0.480084	15585.19588	4126.180694	947.386629	0.137036	0.183545
E07	MMP12	-8.404509	-9.148301	-2.42875	4.459971	5.259679	2.86811	338.851418	567.431106	5.384269	0.452094	0.267912
E08	MMP13	-5.871958	-6.178648	-1.086642	1.716866	2.874689	1.257157	58.564642	72.43664	2.123791	0.512067	0.134336
E09	MMP14	-15.766552	-16.094172	-12.514694	0.969764	1.060538	0.777851	55744.90511	69956.56624	5851.918655	0.604596	0.051888
E10	MMP15	-3.982454	-6.028272	-1.38771	3.741264	1.388449	2.452994	15.806584	65.266543	2.61663	0.917809	0.31173
E11	MMP16	-10.985766	-12.249527	-10.429002	0.797603	1.063943	0.474313	2027.892789	4869.395126	1378.612704	0.286794	0.295753
E12	MMP2	-16.110788	-15.081117	-13.645707	0.870195	1.122721	0.572062	70766.92771	34663.20145	12816.43145	0.255231	0.048672
F01	MMP3	-7.176545	-5.442859	-1.613389	7.044101	2.890981	1.294068	144.662248	43.497448	3.059697	0.136021	0.27943
F02	MMP7	-6.404299	-4.725502	-1.400958	6.084153	3.61378	1.650341	84.700544	26.455606	2.640768	0.147786	0.284178
F03	MMP8	-2.87605	-6.876461	-4.550743	4.981464	4.330142	4.209255	7.341372	117.495455	23.437434	0.357553	0.667554
F04	MMP9	-5.513114	-7.691342	-2.680328	1.014128	2.169595	1.121506	45.668062	206.692487	6.410016	0.417166	0.066155
F05	NCAM1	-13.506044	-11.505826	-10.450635	0.890322	1.533271	0.671371	11633.87429	2908.028543	1399.440807	0.280801	0.041961
F06	PECAM1	-11.895802	-11.557976	-8.805716	1.258463	1.067559	1.156273	3810.597903	3015.070755	447.491355	0.614984	0.092131
F07	SELE	-0.947657	-5.605208	0	1.641389	3.734459	0	1.928737	48.678323	1	0.336431	0.285591
F08	SELL	-1.441891	-4.048033	-0.446931	1.334284	2.531997	0.893862	2.716767	16.541673	1.363137	0.348881	0.255964
F09	SELP	-6.020363	-6.355765	-0.858262	2.324221	3.051896	0.838188	64.909737	81.898509	1.812853	0.61074	0.216361
F10	SGCE	-12.506822	-10.61093	-9.924485	0.916708	0.717987	0.637845	5820.073538	1563.897545	971.779205	0.01635	0.048187
F11	SPARC	-18.630126	-18.06768	-16.508394	0.719176	0.836812	0.463725	405720.2166	274734.7463	93222.70467	0.3657	0.029925
F12	SPG7	-12.096634	-12.607678	-9.593139	1.664787	1.236187	0.611873	4379.752611	6241.505243	772.365011	0.783913	0.18205
G01	SPP1	-3.592553	-7.827117	-4.420205	3.215522	2.436749	0.732158	12.063305	227.08947	21.40989	0.248186	0.671331
G02	TGFBI	-18.979475	-16.347295	-16.102953	0.821047	0.683363	0.340209	516881.8435	83373.12056	70383.67626	0.005116	0.03283
G03	THBS1	-18.895291	-17.188013	-16.837352	0.709689	0.996772	0.591227	487583.7997	149316.3743	117097.6015	0.016816	0.034505
G04	THBS2	-8.984129	-7.784198	-7.609522	1.705503	4.017015	0.789663	506.39827	220.433265	195.296462	0.701506	0.255252
G05	THBS3	-11.268987	-10.42003	-8.278605	1.262715	1.597972	0.825102	2467.761634	1370.06617	310.533478	0.572216	0.105626
G06	TIMP1	-17.636331	-17.941066	-14.352979	0.845264	1.090338	0.400159	203734.4842	251651.2582	20925.55838	0.548278	0.032304
G07	TIMP2	-16.225732	-15.932511	-13.609307	0.867053	0.820629	0.622595	76635.84198	62540.82865	12497.11316	0.661095	0.045676
G08	TIMP3	0	-2.461552	0	0	1.561633	0	1	5.508088	1	0.234126	0
G09	CLEC3B	-5.854566	-4.887308	-0.530733	2.046896	1.292095	1.061466	57.862871	29.59554	1.444663	0.216694	0.188656
G10	TNC	-10.936212	-9.258874	-9.520382	0.905214	1.423214	0.531763	1959.421418	612.63064	734.379639	0.165833	0.101376
G11	VCAM1	-11.134989	-9.773687	-6.854267	1.576138	1.786074	0.775923	2248.876416	875.332562	115.70174	0.454235	0.130614
G12	VTN	-10.648121	-8.798525	-7.528655	2.71635	2.060895	0.855912	1604.736453	445.266551	184.65071	0.325602	0.255782
H01	B2M	-16.889963	-15.791278	-13.438379	0.749422	0.780467	0.777945	121446.6077	56708.52455	11100.8267	0.077064	0.01889
H02	HPRT1	-12.825874	-12.143276	-9.762063	1.130817	0.632085	0.649211	7260.604736	4523.663363	868.30796	0.190665	0.078071
H03	RPL13A	-17.671446	-17.093834	-14.592249	0.860631	0.829765	0.520788	208754.2436	139880.3985	24700.42937	0.366287	0.037106
H04	GAPDH	-19.220776	-18.570045	-15.708745	0.83345	0.486258	0.381422	610984.533	389171.1241	53555.4212	0.149472	0.029543
H05	ACTB	-18.695114	-17.930797	-16.486421	1.039481	0.457166	0.667955	424414.2781	249866.333	91813.67955	0.053767	0.031014
H06	HGDC	-3.280558	-5.119968	-0.534693	5.682093	4.602757	1.069385	9.717315	34.774755	1.448633	0.672845	0.286786
H07	RTC	-3.084823	-2.014751	0	5.34307	3.495817	0	8.484461	4.041109	1	0.343394	0.285591
H08	RTC	0	0	0	0	0	0	1	1	1	0	0
H09	RTC	-4.674883	-4.073009	-2.365292	1.649578	1.509252	0.663199	25.543471	16.830535	5.152568	0.449611	0.142855
H10	PPC	-16.286379	-15.119475	-15.477792	0.84119	1.591932	0.112542	79926.11436	35597.17173	45633.05909	0.718043	0.116588
H11	PPC	-15.682953	-15.272457	-15.415549	0.810605	0.687757	0.137102	52606.48367	39579.26341	43706.15216	0.363672	0.368365
H12	PPC	-15.881651	-14.786024	-15.342314	1.28367	0.655765	0.233715	60374.484	28251.18406	41542.89827	0.074487	0.300268

p-value:

The p values are calculated based on a Student's t-test of the replicate 2^(-Delta Ct) values for each gene in the control group and treatment groups, and p values less than 0.05 are indicated in red.

Up-down regulation (comparing to control) of all genes for Extracellular Matrix and Adhesion Molecules PCR Array (PAHS-013) was listed in below table.

		Up-Down Regulation (comparing to control group)	
		hESC-EC in Matrigel	hESC-EC in PBS
**Position**	**Symbol**	**Fold Regulation**	**Fold Regulation**
A01	ADAMTS1	1.2063	-6.8183
A02	ADAMTS13	-9.7607	-24.2485
A03	ADAMTS8	4.0785	-11.4058
A04	CD44	1.6815	-10.6141
A05	CDH1	-5.3446	-14.423
A06	CNTN1	-1.4071	-1.6942
A07	COL11A1	-3.051	-6.6686
A08	COL12A1	-5.4304	-2.8432
A09	COL14A1	1.519	-5.6423
A10	COL15A1	1.5281	-10.9807
A11	COL16A1	-3.676	-4.9013
A12	COL1A1	-5.0815	-4.7367
B01	COL4A2	-1.8961	-6.1107
B02	COL5A1	-5.0684	-7.458
B03	COL6A1	-2.0278	-8.0417
B04	COL6A2	-1.1955	-4.2833
B05	COL7A1	-8.1975	-24.6557
B06	COL8A1	-5.8805	-5.0149
B07	VCAN	-3.9434	-1.3233
B08	CTGF	-1.7343	-2.6038
B09	CTNNA1	-1.9437	-10.7037
B10	CTNNB1	-1.8429	-7.1544
B11	CTNND1	1.0684	-5.2141
B12	CTNND2	1.4529	-18.8218
C01	ECM1	-2.5295	-12.2278
C02	FN1	-1.5774	-2.0811
C03	HAS1	-2.0726	-7.3207
C04	ICAM1	-1.448	-5.3454
C05	ITGA1	1.2469	-2.1563
C06	ITGA2	3.2508	-4.7397
C07	ITGA3	1.6042	-4.2294
C08	ITGA4	-2.2539	-4.8584
C09	ITGA5	-1.141	-6.5938
C10	ITGA6	-1.8342	-9.1324
C11	ITGA7	1.5445	-10.4983
C12	ITGA8	-2.2998	-4.0953
D01	ITGAL	1.3843	-5.4411
D02	ITGAM	5.3715	-196.3583
D03	ITGAV	1.1448	-3.3757
D04	ITGB1	-2.9542	-5.6206
D05	ITGB2	-1.4824	-18.5512
D06	ITGB3	1.9277	-3.0247
D07	ITGB4	-1.5703	-23.2927
D08	ITGB5	1.4687	-6.2317
D09	KAL1	-1.5474	-1.7215
D10	LAMA1	-1.638	-5.7085
D11	LAMA2	1.3169	-3.6764
D12	LAMA3	4.4775	1.4929
E01	LAMB1	-1.5811	-4.2604
E02	LAMB3	4.2326	-4.411
E03	LAMC1	-1.9341	-7.2838
E04	MMP1	4.6455	-12.9063
E05	MMP10	-5.5909	-16.8065
E06	MMP11	-3.7771	-16.4507
E07	MMP12	1.6746	-62.9336
E08	MMP13	1.2369	-27.5755
E09	MMP14	1.2549	-9.5259
E10	MMP15	4.1291	-6.0408
E11	MMP16	2.4012	-1.471
E12	MMP2	-2.0416	-5.5216
F01	MMP3	-3.3258	-47.2799
F02	MMP7	-3.2016	-32.0742
F03	MMP8	16.0046	3.1925
F04	MMP9	4.526	-7.1245
F05	NCAM1	-4.0006	-8.3132
F06	PECAM1	-1.2639	-8.5155
F07	SELE	25.2384	-1.9287
F08	SELL	6.0887	-1.993
F09	SELP	1.2617	-35.8053
F10	SGCE	-3.7215	-5.9891
F11	SPARC	-1.4768	-4.3522
F12	SPG7	1.4251	-5.6706
G01	SPP1	18.8248	1.7748
G02	TGFBI	-6.1996	-7.3438
G03	THBS1	-3.2654	-4.1639
G04	THBS2	-2.2973	-2.593
G05	THBS3	-1.8012	-7.9468
G06	TIMP1	1.2352	-9.7362
G07	TIMP2	-1.2254	-6.1323
G08	TIMP3	5.5081	1
G09	CLEC3B	-1.9551	-40.0529
G10	TNC	-3.1984	-2.6681
G11	VCAM1	-2.5692	-19.4368
G12	VTN	-3.604	-8.6907

Fold Change & Fold Regulation:

Fold-Change (2^(- Delta Delta Ct)) is the normalized gene expression (2^(- Delta Ct)) in the Test Sample divided the normalized gene expression (2^(- Delta Ct)) in the Control Sample.	Fold-Regulation represents fold-change results in a biologically meaningful way. Fold-change values greater than one indicate a positive- or an up-regulation, and the fold-regulation is equal to the fold-change.	Fold-change values less than one indicate a negative or down-regulation, and the fold-regulation is the negative inverse of the fold-change.	Fold-change and fold-regulation values greater than 2 are indicated in red; fold-change values less than 0.5 and fold-regulation values less than -2 are indicated in blue.

Apoptosis PCR Array (PAHS-012) full gene list and data analysis results can be found in below table:

		AVG Delta(Ct) (Ct(GOI) - Ave Ct(HKG))			Standard Deviation			2^(-Avg.(Delta(Ct))			p-value (comparing to control group)	
Position	Symbol	Control Group	hESC-EC in Matrigel	hESC-EC in PBS	Control Group	hESC-EC in Matrigel	hESC-EC in PBS	Control Group	hESC-EC in Matrigel	hESC-EC in PBS	hESC-EC in Matrigel	hESC-EC in PBS
A01	ABL1	-0.888436	-0.713069	0.375223	0.349671	0.159924	0.148795	1.851168	1.639288	0.770986	0.423374	0.009296
A02	AKT1	-2.584394	-2.00164	-1.108015	0.306985	0.038823	0.262582	5.997636	4.004551	2.155488	0.040236	0.004126
A03	APAF1	0.228563	-0.193723	0.045596	0.073753	0.051448	0.039787	0.853485	1.143711	0.968889	0.000289	0.010324
A04	BAD	-1.053815	-0.424954	0.73989	0.198836	0.33143	0.022343	2.076012	1.34253	0.598785	0.027114	0.000324
A05	BAG1	4.27157	4.539592	5.449577	0.014483	0.301249	0.891869	0.051776	0.042998	0.022883	0.139408	0.030752
A06	BAG3	-2.842268	-3.073461	-1.382181	0.180195	0.111483	0.171314	7.171464	8.417906	2.606621	0.105211	0.000411
A07	BAG4	0.341875	0.37544	0.411997	0.135848	0.237109	0.032531	0.789015	0.77087	0.751582	0.868374	0.414132
A08	BAK1	-1.069297	-0.512587	1.383746	0.183111	0.210009	0.033358	2.09841	1.426606	0.383223	0.015073	0.000112
A09	BAX	-3.768599	-3.239924	-2.641491	0.240497	0.112134	0.033644	13.628914	9.447444	6.239763	0.027386	0.002558
A10	BCL10	-0.574375	-0.619755	0.65515	0.156845	0.374731	0.678018	1.489032	1.536615	0.635009	0.750094	0.008782
A11	BCL2	7.202523	6.319053	7.720989	0.062071	0.070933	0.039772	0.006789	0.012525	0.00474	0.000014	0.000103
A12	BCL2A1	4.267039	4.64745	4.948842	0.248275	0.126763	0.13784	0.051939	0.0399	0.032378	0.074246	0.015107
B01	BCL2L1	-0.944385	-0.943756	-0.34013	0.026099	0.194386	0.144144	1.924369	1.92353	1.265871	0.937251	0.00018
B02	BCL2L10	9.246481	10.55531	9.963087	1.532249	0.279584	0.069694	0.001646	0.000665	0.001002	0.20487	0.291874
B03	BCL2L11	3.943795	5.406137	4.469434	0.371462	0.43994	0.177178	0.064983	0.023583	0.04514	0.010918	0.095218
B04	BCL2L2	0.129155	0.282443	0.819579	0.476809	0.306737	0.067044	0.914367	0.822198	0.566607	0.579005	0.08871
B05	BCLAF1	0.338985	0.237076	0.912275	0.014767	0.57231	0.408419	0.790597	0.848463	0.531347	0.592107	0.027687
B06	BFAR	-0.733665	0.112038	0.74813	0.398974	0.470829	0.931151	1.662859	0.92528	0.595375	0.065404	0.037154
B07	BID	0.377378	0.997145	1.711664	0.085902	0.092559	0.040282	0.769835	0.500991	0.305308	0.000348	0.000013
B08	BIK	7.256471	9.066301	9.942599	0.612334	0.494648	0.444701	0.00654	0.001865	0.001016	0.046152	0.025827
B09	NAIP	2.563278	1.674734	1.805401	0.241562	0.204984	0.039009	0.169191	0.313224	0.286102	0.003033	0.001232
B10	BIRC2	-0.233644	-0.603032	0.101234	0.196483	0.107956	0.266723	1.175801	1.518905	0.932235	0.028817	0.123942
B11	BIRC3	7.246307	10.594267	7.655637	0.770871	0.270541	1.111872	0.006586	0.000647	0.004959	0.027397	0.608445
B12	XIAP	0.422274	0.760514	1.154403	0.104965	0.188642	0.22408	0.746248	0.590286	0.449252	0.028261	0.001634
C01	BIRC6	2.145095	1.341042	1.881747	0.587401	0.208661	0.102304	0.22608	0.394735	0.271355	0.051694	0.600478
C02	BIRC8	8.378731	10.594267	10.221997	0.259841	0.270541	0.384042	0.003004	0.000647	0.000837	0.000639	0.001128
C03	BNIP1	0.881109	1.593124	2.410029	0.152876	0.264916	0.089591	0.54295	0.331453	0.188152	0.005049	0.000147
C04	BNIP2	-0.721346	-0.702068	-0.210049	0.063788	0.037211	0.28825	1.648719	1.626835	1.156727	0.65331	0.012878
C05	BNIP3	-1.90053	-1.552793	-0.494917	0.247209	0.25229	0.271881	3.733503	2.933846	1.409239	0.139111	0.002103
C06	BNIP3L	-2.264553	-2.296034	-1.581215	0.159451	0.093839	0.046943	4.805055	4.911057	2.992217	0.805583	0.002224
C07	BRAF	3.019454	2.64962	3.471713	0.129244	0.193385	0.197509	0.123326	0.159362	0.090138	0.028209	0.014675
C08	NOD1	1.938935	2.379166	3.005515	0.312647	0.098345	0.35492	0.260809	0.19222	0.124523	0.088628	0.014243
C09	CARD6	2.059039	2.502707	3.026835	0.18591	0.136233	0.441229	0.239976	0.176445	0.122696	0.023171	0.00831
C10	CARD8	2.557835	1.973189	3.309624	0.2803	0.335908	0.181974	0.16983	0.254689	0.100856	0.048012	0.018101
C11	CASP1	1.309849	1.799943	2.381479	0.199425	0.048849	0.236704	0.403363	0.287186	0.191913	0.016182	0.002012
C12	CASP10	6.25077	6.099512	7.297918	0.112477	0.762602	0.99118	0.013132	0.014584	0.006355	0.465819	0.028085
D01	CASP14	9.011841	10.455862	10.221997	1.161258	0.132611	0.384042	0.001937	0.000712	0.000837	0.147435	0.178852
D02	CASP2	0.893413	0.344001	1.508943	0.083599	0.241363	0.177532	0.538339	0.787854	0.351369	0.010157	0.001209
D03	CASP3	-0.707704	-0.472589	0.246678	0.148125	0.040493	0.054693	1.633203	1.387598	0.842835	0.055712	0.000524
D04	CASP4	-1.374507	-1.011112	-0.079594	0.377994	0.082383	0.091769	2.592794	2.015464	1.056721	0.177604	0.011023
D05	CASP5	9.063917	8.894709	8.724629	1.284571	1.561928	0.211795	0.001868	0.002101	0.002364	0.78547	0.950438
D06	CASP6	-0.702997	-0.223139	0.337369	0.794011	0.040929	0.095945	1.627883	1.167271	0.791483	0.297858	0.126793
D07	CASP7	-0.355884	-0.594515	0.227245	0.086187	0.149234	0.170411	1.279769	1.509965	0.854265	0.042754	0.001555
D08	CASP8	2.494878	2.643601	4.162148	0.077247	0.162459	0.601175	0.177405	0.160028	0.055856	0.169546	0.000391
D09	CASP9	0.517573	0.779416	1.525757	0.026404	0.087709	0.024996	0.698546	0.582603	0.347297	0.00153	0
D10	CD40	-0.196603	0.063364	0.62579	0.222096	0.300938	0.242764	1.145997	0.95703	0.648065	0.241968	0.008591
D11	CD40LG	5.970926	9.111766	10.221997	0.406527	1.282691	0.384042	0.015943	0.001808	0.000837	0.00363	0.002129
D12	CFLAR	1.272693	1.130925	1.901772	0.275107	0.080997	0.370809	0.413886	0.456623	0.267614	0.473408	0.056272
E01	CIDEA	7.029019	9.321992	8.348397	0.010005	1.101853	0.540637	0.007657	0.001562	0.003068	0.000084	0.000638
E02	CIDEB	2.394547	2.098496	2.954032	0.596028	0.040219	0.587767	0.190182	0.233502	0.129047	0.542561	0.291598
E03	CRADD	-0.030008	0.442064	1.662774	0.209914	0.355454	0.18837	1.021018	0.736081	0.315831	0.069324	0.00055
E04	DAPK1	7.468482	10.30479	9.96697	0.899068	0.572466	0.762424	0.005646	0.000791	0.000999	0.050264	0.059287
E05	DFFA	0.82091	0.739218	2.355987	0.252031	0.343312	0.658549	0.566085	0.599064	0.195334	0.687196	0.005411
E06	FADD	-0.451889	0.303582	1.163233	0.225019	0.132348	0.303112	1.36783	0.810238	0.446511	0.007646	0.000968
E07	FAS	1.554186	1.583343	1.713882	0.417772	0.234207	0.10923	0.340521	0.333708	0.304839	0.831858	0.479029
E08	FASLG	9.479485	10.594267	10.099051	1.265542	0.270541	0.554891	0.001401	0.000647	0.000912	0.200301	0.334239
E09	GADD45A	0.045727	0.520426	1.921424	0.226553	0.254361	0.48035	0.968801	0.697166	0.263994	0.055412	0.000977
E10	HRK	7.603038	10.424024	10.221997	0.841183	0.145103	0.384042	0.005143	0.000728	0.000837	0.046168	0.050153
E11	IGF1R	-1.461029	-0.932078	-0.213734	0.406344	0.052823	0.428667	2.753047	1.908022	1.159685	0.103602	0.021811
E12	LTA	7.02336	8.026908	8.899947	0.796406	2.235445	0.877088	0.007687	0.003834	0.002093	0.601205	0.065518
F01	LTBR	-2.61428	-1.771374	-1.06069	0.048556	0.179419	0.17963	6.123174	3.413789	2.085929	0.000098	0.000003
F02	MCL1	-3.325213	-3.394606	-2.459868	0.196025	0.189867	0.109161	10.022793	10.516667	5.501664	0.660665	0.002774
F03	NOL3	0.19799	1.21566	1.746718	0.229808	0.124073	0.082189	0.871764	0.430576	0.297979	0.003151	0.000903
F04	PYCARD	-2.065449	-1.086349	-0.087351	0.185518	0.069988	0.264602	4.185643	2.12336	1.062418	0.001388	0.000243
F05	RIPK2	-0.724965	-1.084716	0.239564	0.087726	0.172804	0.089583	1.65286	2.120958	0.847001	0.016192	0.000061
F06	TNF	9.34217	10.594267	9.964348	1.422625	0.270541	0.063153	0.001541	0.000647	0.001001	0.200602	0.310216
F07	TNFRSF10A	3.008613	2.909663	3.770108	0.087733	0.086421	0.978302	0.124256	0.133077	0.073297	0.196097	0.278708
F08	TNFRSF10B	-2.061625	-2.515811	-1.810754	0.014581	0.427327	0.297749	4.174562	5.719189	3.508255	0.114135	0.161262
F09	TNFRSF11B	2.181883	1.496555	2.71855	0.426357	0.140913	0.408687	0.220388	0.354399	0.151927	0.030261	0.175764
F10	TNFRSF1A	1.249346	3.288198	3.959791	0.232094	0.294915	0.40332	0.420639	0.102366	0.064266	0.000615	0.000352
F11	TNFRSF21	-0.227663	0.939437	1.156536	0.493086	0.026895	0.211684	1.170937	0.521436	0.448588	0.036043	0.026847
F12	TNFRSF25	1.78822	1.965759	2.654334	0.112141	0.091847	0.42938	0.289529	0.256004	0.158842	0.079989	0.00658
G01	CD27	8.275976	6.76844	7.269713	0.244228	0.540886	1.076592	0.003226	0.009173	0.00648	0.020713	0.192045
G02	TNFRSF9	7.675499	7.388829	10.184131	2.064151	3.087736	0.4327	0.004892	0.005967	0.00086	0.510678	0.244514
G03	TNFSF10	7.455208	5.359541	6.718542	0.465009	0.780069	1.227405	0.005698	0.024357	0.009495	0.030239	0.187597
G04	CD70	4.617556	4.482898	6.756597	0.171131	0.709424	0.698016	0.040736	0.044721	0.009248	0.545349	0.000303
G05	TNFSF8	8.662309	5.293481	9.906069	1.130601	0.381567	0.861744	0.002468	0.025498	0.001042	0.001441	0.126187
G06	TP53	-2.00126	-1.045643	-0.367882	0.184232	0.175337	0.320369	4.003496	2.064287	1.290457	0.001892	0.000474
G07	TP53BP2	0.836597	1.174726	2.486329	0.123954	0.166854	0.558623	0.559963	0.442968	0.17846	0.02962	0.000542
G08	TP73	7.124113	10.594267	10.221997	0.418244	0.270541	0.384042	0.007168	0.000647	0.000837	0.001569	0.001825
G09	TRADD	-1.155221	-0.175878	0.980655	0.155149	0.133578	0.070269	2.227184	1.129652	0.50675	0.000767	0.000071
G10	TRAF2	2.267642	1.922753	2.134534	0.155928	0.061013	0.30453	0.207669	0.263751	0.227741	0.011857	0.445141
G11	TRAF3	0.390778	-0.082005	0.268459	0.206832	0.330734	0.352779	0.762718	1.058488	0.830206	0.062673	0.552605
G12	TRAF4	-0.559977	-0.175557	-0.449939	0.076541	0.282714	0.052184	1.474246	1.129401	1.365982	0.030927	0.087363
H01	B2M	-5.712706	-4.768024	-3.963532	0.151798	0.059564	0.137231	52.444023	27.246966	15.600629	0.000603	0.000106
H02	HPRT1	-0.808268	-1.082402	-0.019078	0.282728	0.236509	0.090625	1.751108	2.117558	1.013311	0.224978	0.014122
H03	RPL13A	-5.679376	-4.912041	-4.239964	0.117931	0.053274	0.162647	51.24631	30.107285	18.895406	0.000403	0.00007
H04	GAPDH	-7.840598	-7.147966	-5.378315	0.04848	0.054129	0.65212	229.221366	141.824835	41.594319	0.000015	0.000018
H05	ACTB	-8.022811	-6.877721	-5.836853	0.075694	0.068342	0.599331	260.079832	117.598108	57.156774	0.000013	0.00005
H06	HGDC	9.983376	9.11844	9.881658	0.902917	0.077883	0.802075	0.000988	0.001799	0.00106	0.146802	0.935223
H07	RTC	10.664434	10.594267	10.221997	0.217045	0.270541	0.384042	0.000616	0.000647	0.000837	0.710918	0.105362
H08	RTC	10.664434	10.594267	10.221997	0.217045	0.270541	0.384042	0.000616	0.000647	0.000837	0.710918	0.105362
H09	RTC	10.664434	10.594267	10.221997	0.217045	0.270541	0.384042	0.000616	0.000647	0.000837	0.710918	0.105362
H10	PPC	-6.507865	-6.093162	-6.670445	0.10175	0.069305	0.183512	91.00442	88.269142	101.860079	0.0623	0.195602
H11	PPC	-6.010221	-5.658236	-6.231535	0.186416	0.188381	0.1956	64.455027	50.50086	75.141322	0.06155	0.190572
H12	PPC	-6.090919	-5.647509	-6.073448	0.317181	0.057227	0.438475	68.163108	50.126747	67.342635	0.085315	0.992465

p-value:

The p values are calculated based on a Student's t-test of the replicate 2^(- Delta Ct) values for each gene in the control group and treatment groups, and p values less than 0.05 are indicated in red.

Up-down regulation (comparing to control) of all genes for apoptosis PCR Array **(**PAHS-012) was listed in below table.

	Up-Down Regulation (comparing to control group)		
		hESC-EC in Matrigel	hESC-EC in PBS
Position	Symbol	Fold Regulation	Fold Regulation
A01	ABL1	-1.1293	-2.401
A02	AKT1	-1.4977	-2.7825
A03	APAF1	1.34	1.1352
A04	BAD	-1.5463	-3.467
A05	BAG1	-1.2042	-2.2626
A06	BAG3	1.1738	-2.7512
A07	BAG4	-1.0235	-1.0498
A08	BAK1	-1.4709	-5.4757
A09	BAX	-1.4426	-2.1842
A10	BCL10	1.032	-2.3449
A11	BCL2	1.8448	-1.4324
A12	BCL2A1	-1.3017	-1.6041
B01	BCL2L1	-1.0004	-1.5202
B02	BCL2L10	-2.4774	-1.6433
B03	BCL2L11	-2.7556	-1.4396
B04	BCL2L2	-1.1121	-1.6138
B05	BCLAF1	1.0732	-1.4879
B06	BFAR	-1.7971	-2.793
B07	BID	-1.5366	-2.5215
B08	BIK	-3.506	-6.4358
B09	NAIP	1.8513	1.691
B10	BIRC2	1.2918	-1.2613
B11	BIRC3	-10.1821	-1.3281
B12	XIAP	-1.2642	-1.6611
C01	BIRC6	1.746	1.2003
C02	BIRC8	-4.6445	-3.5882
C03	BNIP1	-1.6381	-2.8857
C04	BNIP2	-1.0135	-1.4253
C05	BNIP3	-1.2726	-2.6493
C06	BNIP3L	1.0221	-1.6059
C07	BRAF	1.2922	-1.3682
C08	NOD1	-1.3568	-2.0945
C09	CARD6	-1.3601	-1.9559
C10	CARD8	1.4997	-1.6839
C11	CASP1	-1.4045	-2.1018
C12	CASP10	1.1105	-2.0664
D01	CASP14	-2.7208	-2.3136
D02	CASP2	1.4635	-1.5321
D03	CASP3	-1.177	-1.9377
D04	CASP4	-1.2865	-2.4536
D05	CASP5	1.1244	1.2651
D06	CASP6	-1.3946	-2.0567
D07	CASP7	1.1799	-1.4981
D08	CASP8	-1.1086	-3.1761
D09	CASP9	-1.199	-2.0114
D10	CD40	-1.1975	-1.7683
D11	CD40LG	-8.8204	-19.0415
D12	CFLAR	1.1033	-1.5466
E01	CIDEA	-4.9006	-2.4956
E02	CIDEB	1.2278	-1.4737
E03	CRADD	-1.3871	-3.2328
E04	DAPK1	-7.1419	-5.6509
E05	DFFA	1.0583	-2.898
E06	FADD	-1.6882	-3.0634
E07	FAS	-1.0204	-1.1171
E08	FASLG	-2.1656	-1.5364
E09	GADD45A	-1.3896	-3.6698
E10	HRK	-7.0665	-6.1431
E11	IGF1R	-1.4429	-2.374
E12	LTA	-2.0049	-3.6721
F01	LTBR	-1.7937	-2.9355
F02	MCL1	1.0493	-1.8218
F03	NOL3	-2.0246	-2.9256
F04	PYCARD	-1.9712	-3.9397
F05	RIPK2	1.2832	-1.9514
F06	TNF	-2.3819	-1.5392
F07	TNFRSF10A	1.071	-1.6952
F08	TNFRSF10B	1.37	-1.1899
F09	TNFRSF11B	1.6081	-1.4506
F10	TNFRSF1A	-4.1092	-6.5452
F11	TNFRSF21	-2.2456	-2.6103
F12	TNFRSF25	-1.131	-1.8227
G01	CD27	2.8432	2.0087
G02	TNFRSF9	1.2198	-5.6908
G03	TNFSF10	4.2742	1.6663
G04	CD70	1.0978	-4.4047
G05	TNFSF8	10.3304	-2.3681
G06	TP53	-1.9394	-3.1024
G07	TP53BP2	-1.2641	-3.1378
G08	TP73	-11.0821	-8.5616
G09	TRADD	-1.9716	-4.395
G10	TRAF2	1.2701	1.0967
G11	TRAF3	1.3878	1.0885
G12	TRAF4	-1.3053	-1.0793

Fold Change & Fold Regulation:

Fold-Change (2^(- Delta Delta Ct)) is the normalized gene expression (2^(- Delta Ct)) in the Test Sample divided the normalized gene expression (2^(- Delta Ct)) in the Control Sample.	Fold-Regulation represents fold-change results in a biologically meaningful way. Fold-change values greater than one indicate a positive- or an up-regulation, and the fold-regulation is equal to the fold-change.	Fold-change values less than one indicate a negative or down-regulation, and the fold-regulation is the negative inverse of the fold-change.	Fold-change and fold-regulation values greater than 2 are indicated in red; fold-change values less than 0.5 and fold-regulation values less than -2 are indicated in blue.

Supplemental Figures

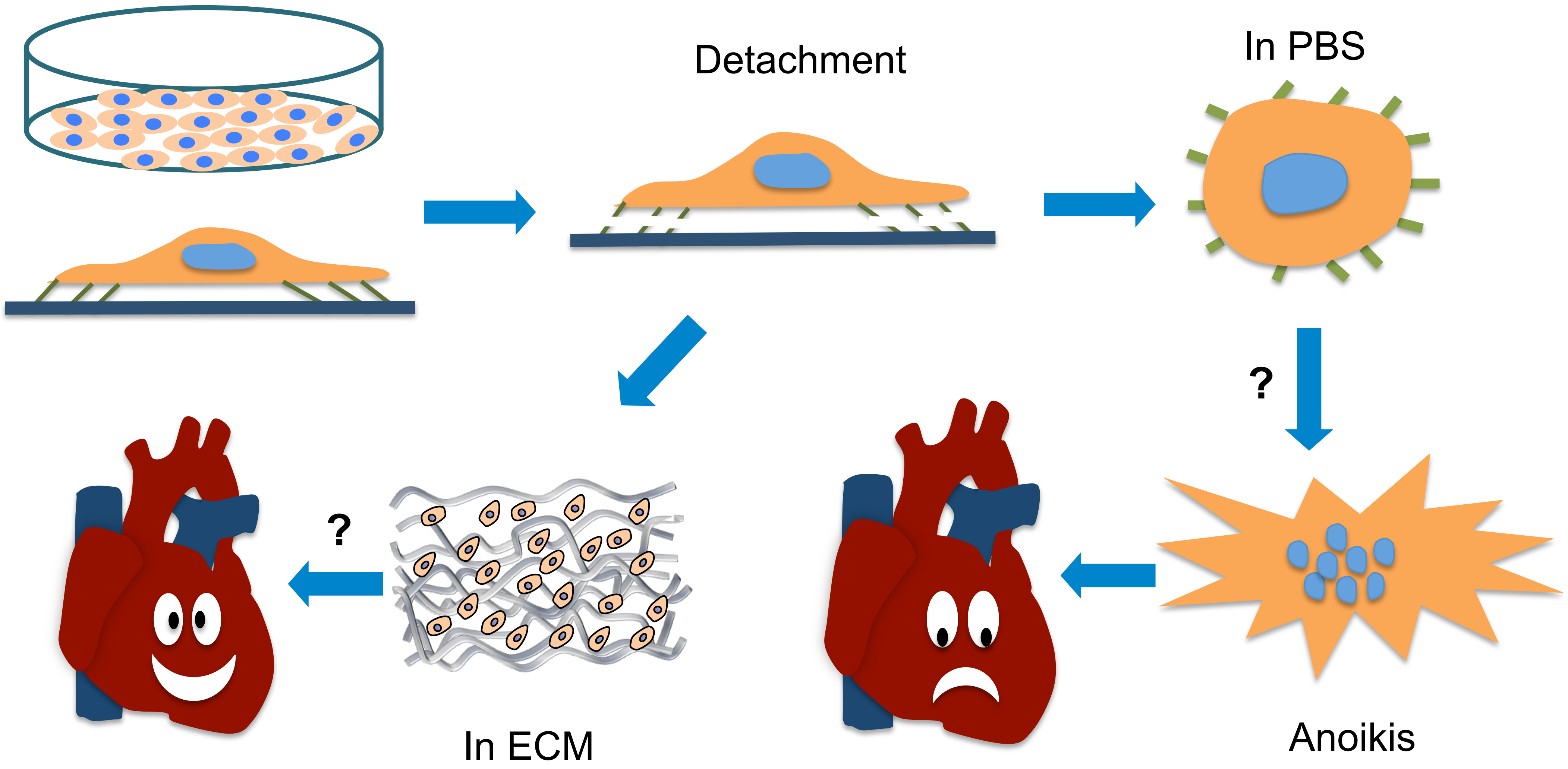

Figure S1. Hypothesis of this study. Enzymatically dispersed cells suspended in PBS will initiate cell anoikis, which affect subsequent cell engraftment. Extracellular matrix (ECM) could increase cell viability and promote heart function recovery. ZL drew this figure with Microsoft PowerPoint.

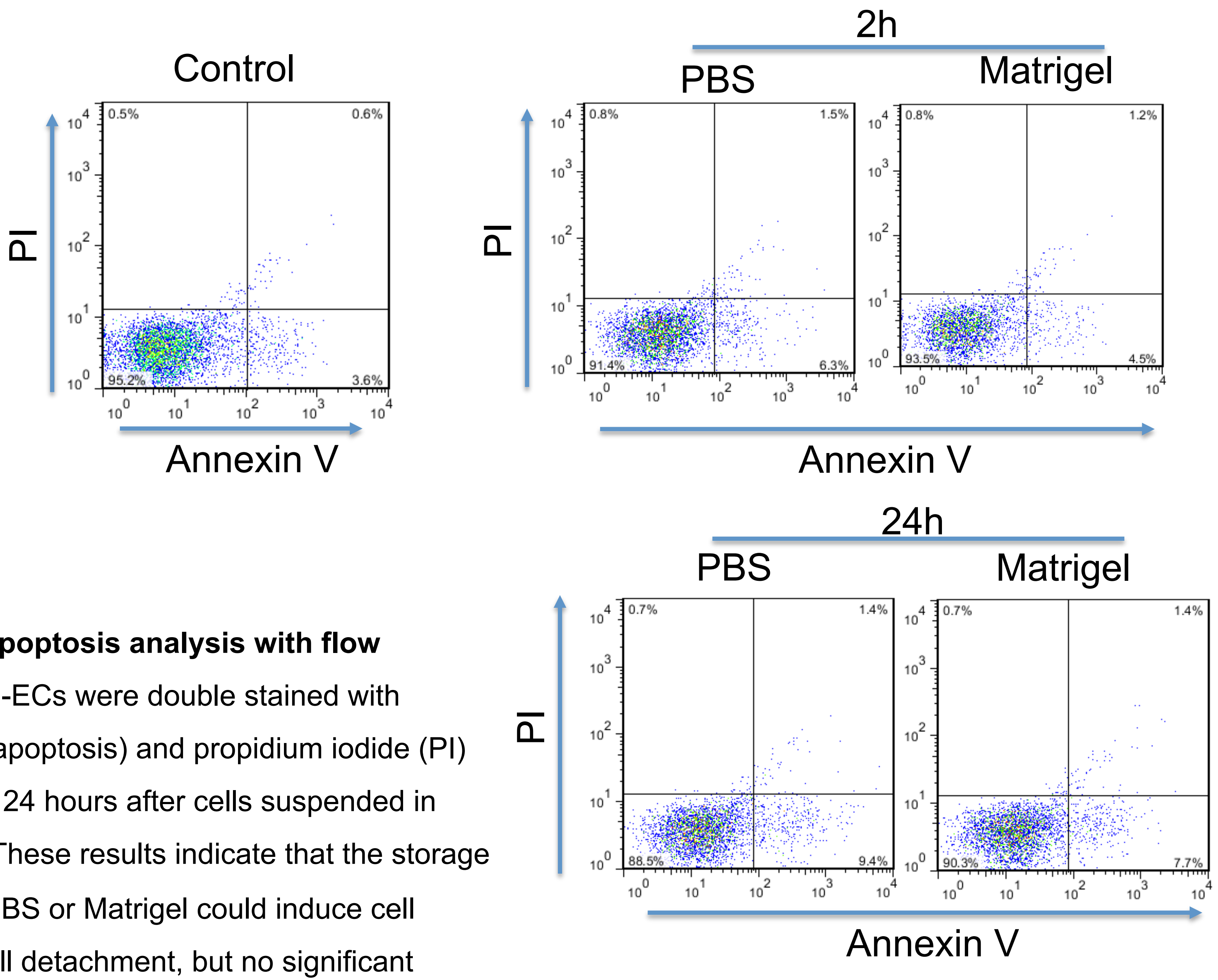

Figure S2: Cell apoptosis analysis with flow cytometry. hESC-ECs were double stained with Annexin V (early apoptosis) and propidium iodide (PI) (cell death) 2 and 24 hours after cells suspended in PBS or Matrigel. These results indicate that the storage of hESC-ECs in PBS or Matrigel could induce cell apoptosis after cell detachment, but no significant difference between two groups

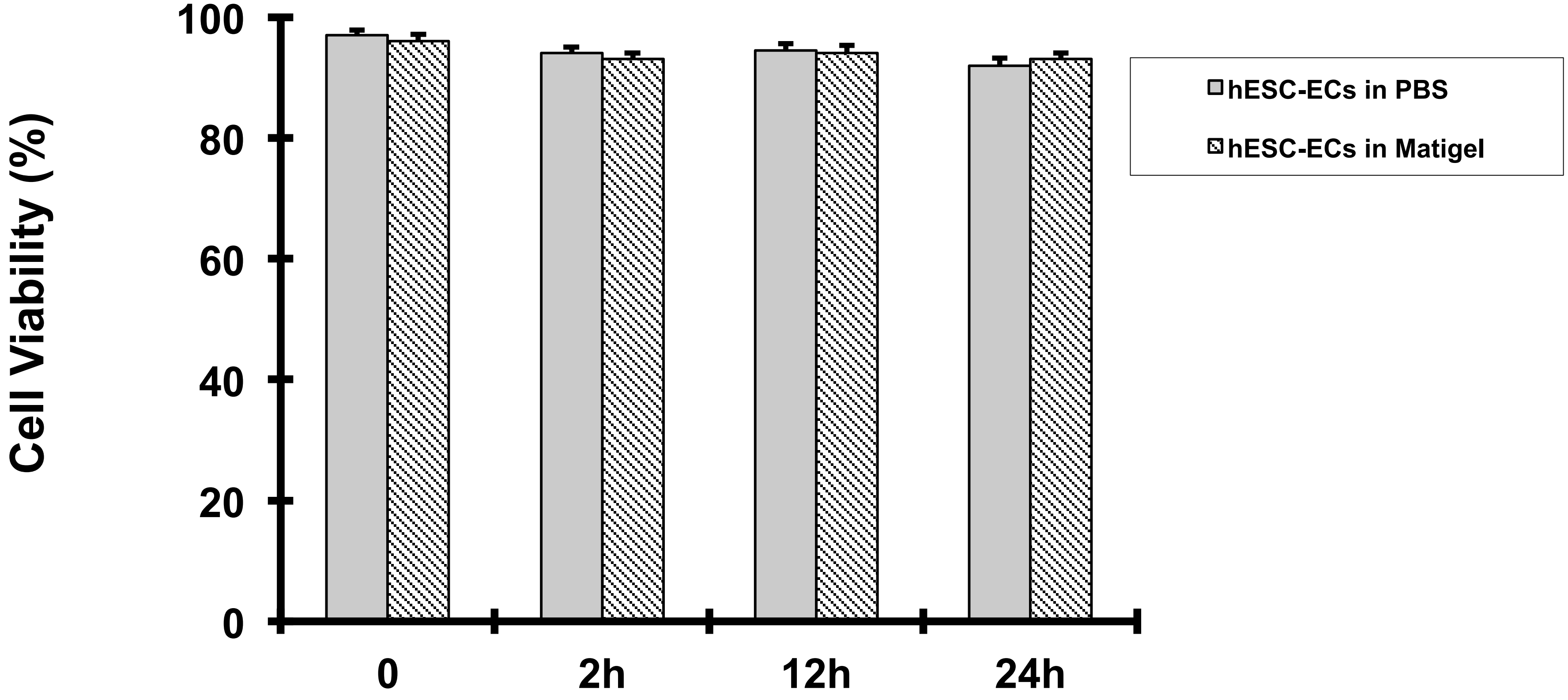

Figure S3: Cell viability assay by trypan blue exclusion. Trypan blue exclusion assay results revealed slightly cell viability decreasing, but no significant difference between hESC-ECs suspended in PBS and Matrigel.

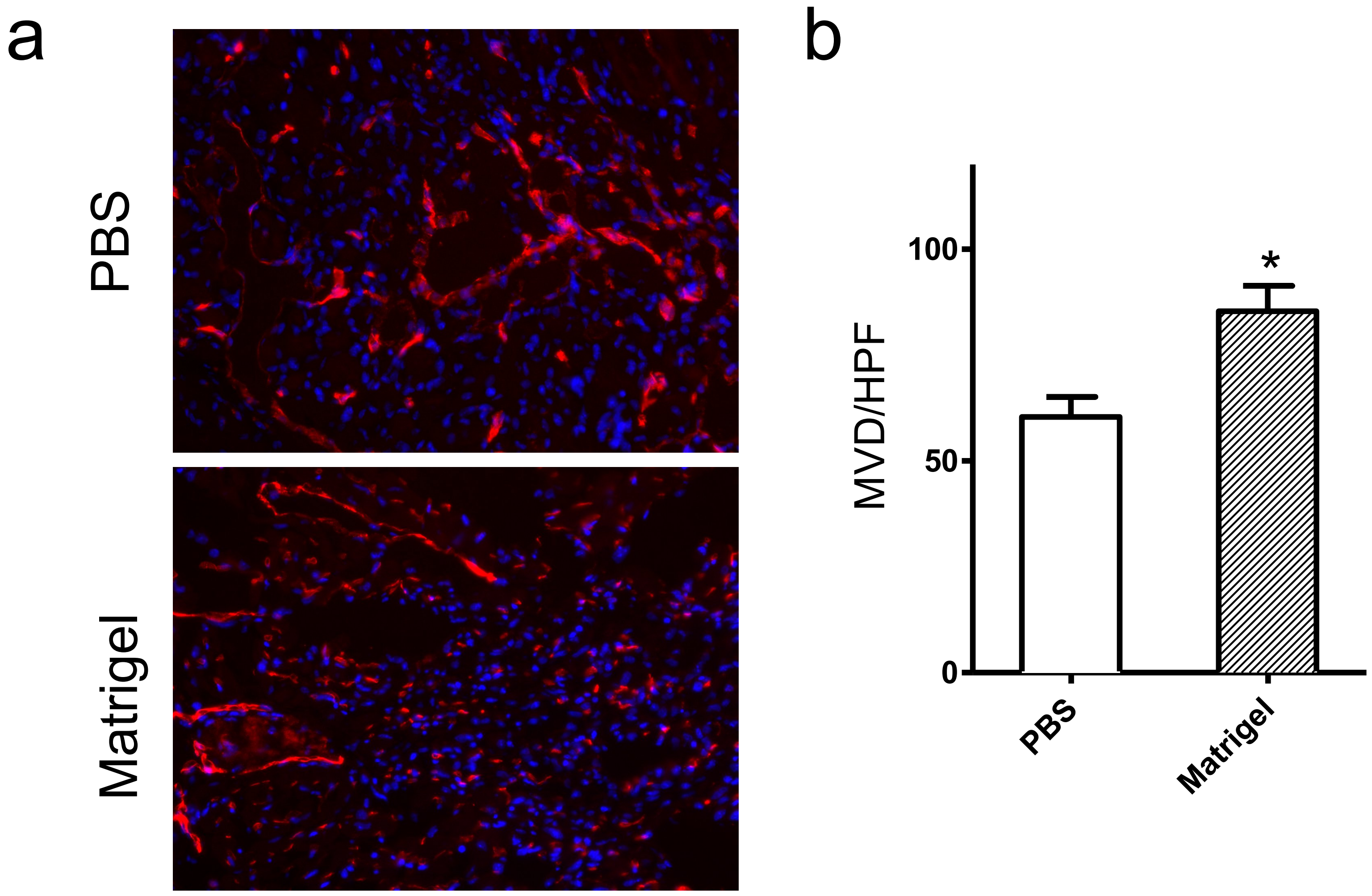

Figure S4: Angiogenesis analysis of Matrigel injection. CD31 staining was done at day 56 after Matrigel only injection. Increased angiogenesis was observed with Matrigel application compared to control. **P*<0.05

Supplemental Tables

Table S1: Functional annotation of the adhesion molecules and ECM genes upregulated of hESC-ECs in PBS compared to control.

Genes Under-Expressed in hESC-EC in PBS vs. Control		
Position	Gene Symbol	Fold Regulation
A01	ADAMTS1	-6.8183
A02	ADAMTS13	-24.2485
A03	ADAMTS8	-11.4058
A04	CD44	-10.6141
A05	CDH1	-14.423
A07	COL11A1	-6.6686
A09	COL14A1	-5.6423
A10	COL15A1	-10.9807
A11	COL16A1	-4.9013
A12	COL1A1	-4.7367
B01	COL4A2	-6.1107
B02	COL5A1	-7.458
B03	COL6A1	-8.0417
B04	COL6A2	-4.2833
B05	COL7A1	-24.6557
B06	COL8A1	-5.0149
B09	CTNNA1	-10.7037
B10	CTNNB1	-7.1544
B11	CTNND1	-5.2141
B12	CTNND2	-18.8218
C01	ECM1	-12.2278
C03	HAS1	-7.3207
C04	ICAM1	-5.3454
C06	ITGA2	-4.7397
C07	ITGA3	-4.2294
C08	ITGA4	-4.8584
C09	ITGA5	-6.5938
C10	ITGA6	-9.1324
C11	ITGA7	-10.4983
C12	ITGA8	-4.0953
D01	ITGAL	-5.4411
D02	ITGAM	-196.3583
D04	ITGB1	-5.6206
D05	ITGB2	-18.5512
D07	ITGB4	-23.2927
D08	ITGB5	-6.2317
D10	LAMA1	-5.7085
E01	LAMB1	-4.2604
E02	LAMB3	-4.411
E03	LAMC1	-7.2838
E04	MMP1	-12.9063
E05	MMP10	-16.8065
E06	MMP11	-16.4507
E07	MMP12	-62.9336
E08	MMP13	-27.5755
E09	MMP14	-9.5259
E10	MMP15	-6.0408
E12	MMP2	-5.5216
F01	MMP3	-47.2799
F02	MMP7	-32.0742
F04	MMP9	-7.1245
F05	NCAM1	-8.3132
F06	PECAM1	-8.5155
F09	SELP	-35.8053
F10	SGCE	-5.9891
F11	SPARC	-4.3522
F12	SPG7	-5.6706
G02	TGFBI	-7.3438
G03	THBS1	-4.1639
G05	THBS3	-7.9468
G06	TIMP1	-9.7362
G07	TIMP2	-6.1323
G09	CLEC3B	-40.0529
G11	VCAM1	-19.4368
G12	VTN	-8.6907
H01	B2M	-10.9403
H02	HPRT1	-8.3618
H03	RPL13A	-8.4514
H04	GAPDH	-11.4085
H05	ACTB	-4.6226
H06	HGDC	-6.7079

Genes Over-Expressed in hESC-EC in PBS vs. Control		
Position	Gene Symbo	Fold Regulation

Table S2: Functional annotation of the adhesion molecules and ECM genes upregulated/downregulated of hESC-ECs+Matrigel compared to control.

Genes Over-Expressed in		
hESC+Matrigel vs. Control Group		
**Position**	**Gene Symbo**	**Fold Regulation**
A03	ADAMTS8	4.0785
D02	ITGAM	5.3715
D12	LAMA3	4.4775
E02	LAMB3	4.2326
E04	MMP1	4.6455
E10	MMP15	4.1291
F03	MMP8	16.0046
F04	MMP9	4.526
F07	SELE	25.2384
F08	SELL	6.0887
G01	SPP1	18.8248
G08	TIMP3	5.5081

Genes Under-Expressed in		
hESC-EC+Matrigel vs. Control Group		
**Position**	**Gene Symbo**	**Fold Regulation**
A02	ADAMTS13	-9.7607
A05	CDH1	-5.3446
A08	COL12A1	-5.4304
A12	COL1A1	-5.0815
B02	COL5A1	-5.0684
B05	COL7A1	-8.1975
B06	COL8A1	-5.8805
E05	MMP10	-5.5909
F05	NCAM1	-4.0006
G02	TGFBI	-6.1996

Table S3: Functional annotation of the adhesion molecules and ECM genes upregulated/downregulated of hESC-ECs in Matrigel compared to hESC-ECs in PBS.

Genes Over-Expressed in hESC-EC in Matrigel vs. hESC-EC in PBS		
Position	Gene Symbol	Fold Regulation
A01	ADAMTS1	8.2248
A03	ADAMTS8	46.5184
A04	CD44	17.8473
A09	COL14A1	8.5705
A10	COL15A1	16.7798
B09	CTNNA1	5.507
B11	CTNND1	5.5706
B12	CTNND2	27.3462
C01	ECM1	4.8341
C06	ITGA2	15.4078
C07	ITGA3	6.7848
C09	ITGA5	5.7791
C10	ITGA6	4.979
C11	ITGA7	16.2142
D01	ITGAL	7.5321
D02	ITGAM	1054.7439
D05	ITGB2	12.5146
D06	ITGB3	5.8307
D07	ITGB4	14.8328
D08	ITGB5	9.1523
D11	LAMA2	4.8414
E02	LAMB3	18.67
E04	MMP1	59.9563
E06	MMP11	4.3553
E07	MMP12	105.3868
E08	MMP13	34.1072
E09	MMP14	11.9545
E10	MMP15	24.943
F01	MMP3	14.2163
F02	MMP7	10.0181
F03	MMP8	5.0132
F04	MMP9	32.2452
F06	PECAM1	6.7377
F07	SELE	48.6783
F08	SELL	12.135
F09	SELP	45.1766
F12	SPG7	8.081
G01	SPP1	10.6068
G05	THBS3	4.412
G06	TIMP1	12.026
G07	TIMP2	5.0044
G08	TIMP3	5.5081
G09	CLEC3B	20.4861
G11	VCAM1	7.5654
H01	B2M	5.1085
H02	HPRT1	5.2097
H03	RPL13A	5.6631
H04	GAPDH	7.2667
H06	HGDC	24.0052

Genes Under-Expressed in hESC-EC in Matrigel vs. hESC-EC in PBS		
Position	Gene Symbol	Fold Regulation

Table S4: Functional annotation of the apoptosis related genes upregulated/downregulated of hESC-ECs in PBS compared to Control.

Genes Over-Expressed in hESC-EC in PBS vs. Control		
Position	Gene Symbol	Fold Regulation

Genes Under-Expressed in hESC-EC in PBS vs. Control		
Position	Gene Symbol	Fold Regulation
A08	BAK1	-5.4757
B08	BIK	-6.4358
D11	CD40LG	-19.0415
E04	DAPK1	-5.6509
E10	HRK	-6.1431
F10	TNFRSF1A	-6.5452
G02	TNFRSF9	-5.6908
G04	CD70	-4.4047
G08	TP73	-8.5616
G09	TRADD	-4.395

Table S5: Functional annotation of the apoptosis related genes upregulated/downregulated of hESC-ECs in Matrigel compared to Control.

Genes Over-Expressed in hESC-EC in Matrigel vs. Control		
**Position**	**Gene Symbol**	**Fold Regulation**
G03	TNFSF10	4.2742
G05	TNFSF8	10.3304

Genes Under-Expressed in hESC-EC in Matrigel vs. Control		
**Position**	**Gene Symbol**	**Fold Regulation**
B11	BIRC3	-10.1821
C02	BIRC8	-4.6445
D11	CD40LG	-8.8204
E01	CIDEA	-4.9006
E04	DAPK1	-7.1419
E10	HRK	-7.0665
F10	TNFRSF1A	-4.1092
G08	TP73	-11.0821

Table S6: Functional annotation of the apoptosis related genes upregulated/downregulated of hESC-ECs in Matrigel compared to hESC-ECs in PBS.

Genes Over-Expressed in hESC-EC in Matrigel vs. hESC-EC in PBS		
Position	Gene Symbol	Fold Regulation
G02	TNFRSF9	6.9418
G04	CD70	4.8356
G05	TNFSF8	24.464

Genes Under-Expressed in hESC-EC in Matrigel vs. hESC-EC in PBS		
Position	Gene Symbol	Fold Regulation
B11	BIRC3	-7.6668
